# Chemotoxicity and Associated Risk Factors in Colorectal Cancer: A Systematic Review and Meta-Analysis

**DOI:** 10.3390/cancers16142597

**Published:** 2024-07-20

**Authors:** Claire J. Han, Xia Ning, Christin E. Burd, Daniel J. Spakowicz, Fode Tounkara, Matthew F. Kalady, Anne M. Noonan, Susan McCabe, Diane Von Ah

**Affiliations:** 1 Center for Healthy Aging, Self-Management and Complex Care, College of Nursing, The Ohio State University, Columbus, OH 43210, USA; mccabe.2@osu.edu (S.M.); vonah.1@osu.edu (D.V.A.); 2 The Ohio State University–James: Cancer Treatment and Research Center, Columbus, OH 43210, USA; 3 Clinical Informatics and Implementation Science Biomedical Informatics (BMI), Computer Science and Engineering (CSE), College of Engineering, The Ohio State University, Columbus, OH 43210, USA; ning.104@osu.edu; 4 Departments of Molecular Genetics, Cancer Biology and Genetics, The Ohio State University, Columbus, OH 43210, USA; christin.burd@osumc.edu; 5 Pelotonia Institute for Immuno-Oncology, Division of Medical Oncology, The Ohio State University, Comprehensive Cancer Center, Columbus, OH 43210, USA; daniel.spakowicz@osumc.edu; 6 Department of Biomedical Informatics, Ohio State University College of Medicine, Columbus, OH 43210, USA; fode.tounkara@osumc.edu; 7 Division of Colon and Rectal Surgery, The Ohio State University–James: Cancer Treatment and Research Center, Columbus, OH 43210, USA; matthew.kalady@osumc.edu; 8 GI Medical Oncology Selection, The Ohio State University–James: Cancer Treatment and Research Center, Columbus, OH 43210, USA; anne.noonan@osumc.edu

**Keywords:** colorectal cancer, chemotoxicity, prevalence, risk factors, review

## Abstract

**Simple Summary:**

Chemotoxicity in colorectal cancer (CRC) patients impacts treatment compliance, survival, and quality of life. While clinician-reported chemotoxicity predicts rehospitalization and survival, a comprehensive understanding is lacking. This systematic review and meta-analysis studied chemotoxicity in CRC patients. The study found that 45.7% of patients experienced overall moderate-to-severe toxicities, with gastrointestinal toxicity (22.9%) and neuropathy or neutropenia (17.9%) being the most common. Risk factors included malnutrition, frailty, impaired immune or hepato-renal functions, short telomere lengths, low gut lactobacillus levels, age, female sex, aggressive chemotherapy, and low quality of life. Age was linked to neutropenia and gastrointestinal toxicity. Older adults had higher overall and gastrointestinal toxicities but lower neutropenia than younger adults. The study underscores the need for close monitoring and management of chemotoxicity in CRC patients. Community contributions have been instrumental in raising awareness about these issues, advocating for patient support, and future research to improve treatment toxicities in CRC.

**Abstract:**

Background: Colorectal cancer (CRC) patients experience multiple types of chemotoxicity affecting treatment compliance, survival, and quality of life (QOL). Prior research shows clinician-reported chemotoxicity (i.e., grading scales or diagnostic codes) predicts rehospitalization and cancer survival. However, a comprehensive synthesis of clinician-reported chemotoxicity is still lacking. Objectives: We conducted a systematic review and meta-analysis to determine chemotoxicity’s prevalence and risk factors in CRC. Methods: A systematic search from 2009 to 2024 yielded 30 studies for review, with 25 included in the meta-analysis. Results: Pooled prevalences of overall, non-hematological, and hematological moderate-to-severe toxicities were 45.7%, 39.2%, and 25.3%, respectively. The most common clinician-reported chemotoxicities were gastrointestinal (GI) toxicity (22.9%) and neuropathy or neutropenia (17.9%). Significant risk factors at baseline were malnutritional status, frailty, impaired immune or hepato-renal functions, short telomere lengths, low gut lactobacillus levels, age, female sex, aggressive chemotherapy, and low QOL. Age was associated with neutropenia (β: −1.44) and GI toxicity (β:1.85) (*p*-values < 0.01). Older adults (>65 y.o.) had higher prevalences of overall (OR: 1.14) and GI (OR: 1.65) toxicities, but a lower prevalence of neutropenia (OR: 0.65) than younger adults (*p*-values < 0.05). Conclusions. Our findings highlight the importance of closely monitoring and managing chemotoxicity in CRC patients receiving chemotherapy.

## 1. Introduction

Colorectal cancer (CRC) is the fourth most common type of cancer and the second leading cause of cancer-related deaths in the United States (U.S.) when men and women are combined [1,2]. By 2030, the number of new CRC cases is expected to reach around 170,968, marking a 17.3% increase from the total cases in 2020. Additionally, CRC is predicted to result in approximately 64,553 deaths, reflecting a 22.2% rise from 2020 [1,2]. CRC is frequently diagnosed in younger (age < 50 years old) (average 20% of diagnoses in 2019) as well as in older patients, with nearly half of the cases being diagnosed in individuals who are 70 years old or older [3]. While surgery is the primary modality of management for CRC in patients with non-metastatic disease (stages I–III), approximately 70% of CRC patients receive chemotherapy (primarily 5-fluorouracil [5-FU]-based agents) as the most common standard adjuvant cancer treatment after undergoing CRC surgery [4,5,6]. 

Chemotherapy can enhance survival rates in both young-onset and older CRC patients, but it can also cause serious toxicities and exacerbate existing conditions [7]. CRC patients receiving chemotherapy frequently develop chemotoxicity, i.e., the harmful or adverse effects of chemotherapy drugs on healthy cells and tissues in the body and related side effects [7]. These toxicities are measured by both patients and clinicians, with a high incidence of severe events occurring within the first two months of treatment initiation and persisting for months or years [7]. Moreover, patient-reported outcomes highlight the enduring physical and psychosocial symptom distress and functional impairment faced by CRC patients receiving chemotherapy, all of which detrimentally affect health-related quality of life (HRQoL) [8]. 

Chemotoxicity varies across cancer types due to distinct pathological features and treatment approaches [9]. In CRC, gastrointestinal (GI) toxicity is particularly prevalent, largely because the treatments target tissues within the GI tract [5,10,11,12]. Between 50 and 80% of CRC patients with frequent and severe GI symptoms prior to or during chemotherapy experience persistent GI symptoms for months or even years post-chemotherapy [5,10,11,12]. Chemotherapeutic agents can induce a range of pro-inflammatory responses, leading to histopathological changes in the intestinal lining [13]. This inflammation can manifest as diarrhea, abdominal pain, nausea/vomiting, and bloating [13,14]. Moreover, the combination of surgery and radiation can exacerbate these symptoms [13]. Long-term intestinal injury may result in severe GI tract damage, such as strictures, stenosis with obstruction, fistulas, and bowel perforation, which are linked to systemic health problems like sepsis and malnutrition [15]. 

These toxicities are significant concerns in CRC, leading to dose reduction, increased mortality or recurrence rates, treatment non-compliance, emergency room visits, and hospitalization [5,12]. Early identification and management are key to allow treatment to continue as planned or at a lower dose if required [9]. Therefore, when planning chemotherapy, weighing the potential risks against the benefits is crucial [5,12]. Understanding the major types and risk factors of chemotoxicity in CRC is necessary for effective management [16,17,18]. Several risk factors of chemotoxicity have been identified, such as patient and clinical factors (e.g., age, body mass index [BMI], and types and doses of chemotherapy) [7,19]. However, the results of current studies on chemotoxicity in CRC patients [7,16,17,18] have been mixed with several limitations, such as small sample sizes, varied measures, and differences in cancer sites, stages, chemotherapy types, and patient characteristics, which have led to no definitive factors associated with chemotoxicity in CRC. These limitations underscore the need for a comprehensive understanding of chemotoxicity, particularly its types and potential risk factors in CRC patients.

Current studies on chemotoxicity in CRC mainly focus on patient-reported outcomes (PROs) [5,12] and cancer treatment outcomes (e.g., response rates and cancer death) [20,21,22]. In previous studies comparing adverse events between PROs versus clinician-reported outcomes (e.g., grading scales, diagnostic codes, or progress notes) in lung cancer [23], and mixed cancer types [24], patients generally reported symptoms earlier and more frequently than clinicians, had a higher incidence and severity of subjective toxicities, and had poor to modest agreement with clinician-reported values [23,24]. Previous studies have demonstrated that PROs were more strongly associated with daily health status (e.g., HRQoL and distress), while clinician-reported chemotoxicity was more strongly associated with future rehospitalization and survival [23,24]. However, the overall prevalence and associated risk factors of chemotoxicity assessed by clinicians remain unsynthesized, leading to inconclusive evidence [16,17,18].

A comprehensive assessment is needed to bridge research gaps and optimize chemotoxicity management in CRC, considering various approaches, including clinician-graded evaluations. To provide an up-to-date overall consensus on clinician-reported chemotoxicity, this systematic review and meta-analysis aims to investigate the characteristics of chemotoxicity and quantitatively analyze the pooled prevalence of chemotoxicity (i.e., what types of chemotoxicity) according to clinician-reported assessments and the risk factors that predict its occurrence in CRC patients. 

## 2. Materials and Methods

### 2.1. Search Strategies and Data Sources

Following the guidelines of the Preferred Reporting Items for Systematic Reviews and Meta-Analyses (PRISMA) [25], we conducted a systematic review of the literature to integrate the findings of quantitative studies (Supplementary Table S1). The current review was not registered with the Prospective Register of Systematic Reviews (PROSPERO). Our review encompassed seven electronic databases: CINAHL, Medline via PubMed, Cochrane Library (Review and CENTRAL), EMBASE, PsycINFO, and Web of Science. The focus of our research was on the symptom experiences of CRC patients after receiving chemotherapy. Through the use of MeSH terms and manual searches, we examined keywords using Boolean operators (AND, OR) to construct the search strategies: (“colorectal cancer” OR “colon cancer” OR “rectal cancer” OR “CRC” OR “colorectal neoplasms” OR “colorectal adenocarcinoma”) AND (“chemotherapy” OR “chemotoxicity” OR “chemotherapeutic agents” OR “antineoplastic agents”) AND (“risk factors” OR “risk factors” OR “predictive models” OR “prognostic factors” OR “response prediction”) AND (“symptom*” OR “adverse event*” “side effect*”) (details in Supplementary Table S2). Additionally, we identified other relevant studies by reviewing the references cited in the obtained published studies. The full, reproducible search strategies for all included databases are located in Supplementary Table S2. 

### 2.2. Inclusion and Exclusion Criteria

To be included in our review, studies had to meet the following criteria: (a) be published within the last 15 years, from January 2009 to March 2024, to reflect the most up-to-date evidence on a topic; (b) report chemotoxicity reported by clinicians after chemotherapy using quantitative measures; (c) examine patients with CRC receiving chemotherapy as a primary treatment, including but not limited to conventional chemotherapy regimens, targeted therapy, adjuvant or palliative chemotherapy; (d) be based on original or experimental data; (e) examine patients who were 18 years or older and had CRC; and (f) be published in English. 

Studies were excluded if they (a) presented only qualitative results; (b) were not published in English; (c) were review papers, editorials, meta-syntheses, theory-based works, dissertations, or case studies; (d) included patient-reported symptoms as primary outcomes; (e) reported only cancer survival rates; or (f) included CRC patients receiving radiotherapy or surgery as a primary treatment. We also reviewed and included eligible studies from the references of previous reviews, focusing on nutritional status and chemotoxicity in CRC [26,27]. There were no restrictions regarding the timing of chemotoxicity assessment after chemotherapy, whether during the recovery or long-term survivorship phases. This comprehensive approach ensured that all relevant literature was included in our review.

### 2.3. Study Selections and Screening

Two authors (CH and SM) independently evaluated the collected articles based on predetermined criteria, including examining titles, abstracts, and full-text articles. Any discrepancies were addressed through discussion.

### 2.4. Data Extraction and Data Synthesis

Data extraction involved the retrieval of variables from the included studies, such as publication year, study design, country of research, research setting, sample size, participant demographic and cancer-related clinical characteristics, methods of measuring chemotoxicity, risk factors for chemotoxicity, and any impact of chemotoxicity, as well as the primary results and conclusions of the studies. The first author extracted the data using standardized forms, which were then verified by the other authors for accuracy and completeness. Any discrepancies were resolved through consensus-building discussions with a third author. The extracted data were synthesized and presented using narrative descriptions and descriptive statistics.

### 2.5. Methodological Quality Appraisal

Two authors (C.H. and S.M.) evaluated the methodological integrity of each article using the Critical Appraisal Skills Program (CASP) checklists [28]. These checklists—which include 12 questions for observational cohort studies and 11 questions for randomized controlled trials—aim to scrutinize the robustness of the research and the likelihood of bias from the investigator [28]. Each question can be answered with “Yes”, “Can’t tell”, or “No.” However, there is no universally accepted standard for assessing an article’s quality using these checklists. We used a commonly applied arbitrary cut-off in critical appraisal, deeming an article as “high” quality if it satisfied at least 80% of the checklist criteria, “low” quality if it satisfied 50% or less, and “medium” quality if it satisfied more than 50% but less than 80% of the criteria [28]. If the two authors could not agree, a third author provided arbitration (Supplementary Table S3).

### 2.6. Meta-Analytical and Statistical Methods

#### 2.6.1. Publication Bias

We examined funnel plots to visually assess the asymmetry of values of chemotoxicity prevalences (Begg’s test and Egger’s test to test the asymmetry statistically). Heterogeneity was assessed using Q statistics, which measure the squared variance. A *p*-value of less than 0.05 was considered statistically significant, indicating the presence of heterogeneity [29]. Additionally, I^2^ statistics were used to assess heterogeneity, with values below 25.0% indicating no heterogeneity and values above 75.0% indicating high or extreme heterogeneity [29]. We reported results from the random effects, which was appropriate in the presence of heterogeneity among studies in our study. 

#### 2.6.2. Pooled Estimations

A meta-analysis was conducted to combine data from multiple studies to obtain more accurate estimations of the prevalence (i.e., pooled prevalence) of chemotoxicity after chemotherapy and the relationships of risk factors with chemotherapy. This approach enhanced the statistical power compared to analyzing individual studies alone [30]. Only studies that provided information on the prevalence (percentage of research participants reporting at least moderate-to-severe toxicity during or after chemotherapy) with/without examining the risk factors of chemotoxicity were included in our meta-analysis. Furthermore, the pooled estimates for prevalence and/or associations between potential risk factors and chemotoxicity were only analyzed if they were present in at least two studies per each subgroup of chemotoxicity. The pooled prevalence of chemotoxicity was calculated by extracting proportions from all included studies, using weighted mean and standard errors to establish a 95% confidence interval (CI). Additionally, the pooled association between risk factors and chemotoxicity was determined using adjusted/unadjusted Beta coefficients or Odd Ratios (ORs) in meta-regressions or F statistics in a meta-analysis of variance (ANOVA) tests. Adjusted regression models included prevalent cancer stages, metastatic status, cancer types, primary types of chemotherapy regimens, sample size (group categories), and sex group variables (female versus male prevalence). Forest plots were utilized to present the pooled mean prevalences of chemotoxicity, and associations of risk factors with chemotoxicity, along with corresponding 95% CIs. Subgroup analyses were conducted per chemotoxicity subgroups (e.g., hematological, non-hematological, and GI toxicity). A two-sided *p*-value of less than 0.05 was considered statistically significant. For the meta-analysis, the statistical analyses, including funnel and forest plots, were conducted using Comprehensive Meta-Analysis (version 2.2.050; Biostat, Engelwood, NJ, USA). Descriptive statistical analysis was performed using the Statistical Package for the Social Sciences version 23.0 (SPSS, Inc., Chicago, IL, USA).

## 3. Results

### 3.1. Search Results and Methodological Quality Evaluation

**Figure 1:**
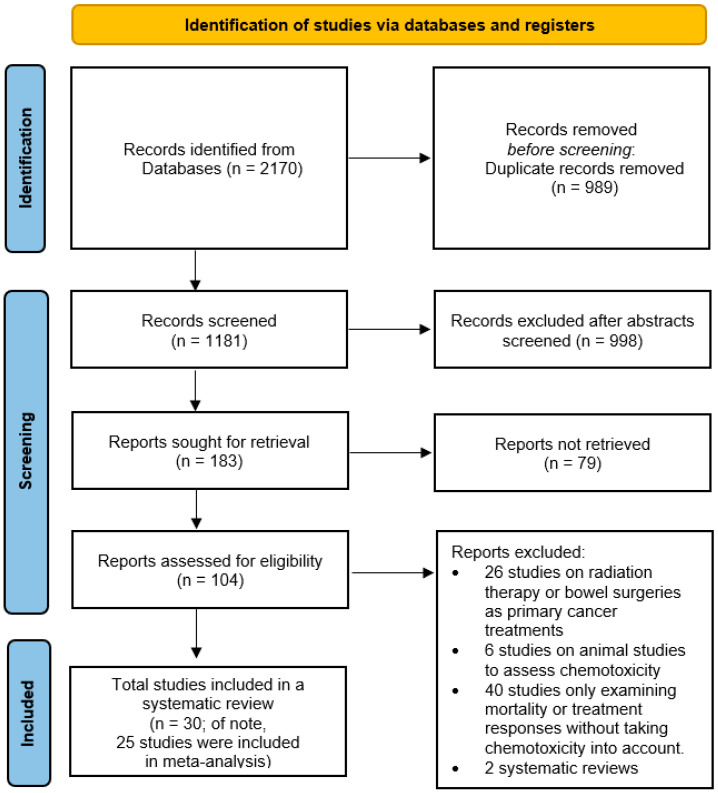
The PRISMA 2020 flow diagram.

The search methods resulted in 1181 articles, excluding duplicates. A review of titles and abstracts narrowed this to 104 relevant studies, with 30 [7,31,32,33,34,35,36,37,38,39,40,41,42,43,44,45,46,47,48,49,50,51,52,53,54,55,56,57,58,59] selected for final analysis after assessing full-text articles. Among the 30 studies included in this review, 5 studies [37,38,39,43,48] were excluded from the meta-analyses due to unavailable chemotoxicity prevalence data or data that were not comparable for inclusion in the meta-analyses. The evaluation of the 30 quantitative studies using CASP tools is detailed in Supplementary Table S3. The inter-rater agreement between authors was 95.8%, and no studies were excluded for low quality. The literature search process is illustrated in Figure 1, and details for the 30 studies are provided in Table 1 and Table 2 and Supplementary Table S4. 

### 3.2. Study Characteristics

The study characteristics reveal that among the 30 studies examined (Table 1 and Table 2), the majority of studies had observational cohort study designs (n = 24 for prospective study design [7,31,32,33,34,36,37,38,39,40,41,42,44,45,46,47,48,51,52,53,54,57,58,59], n = 5 for retrospective study design [35,43,49,55,56], while 1 study used a randomized control trial (RCT) [50].) Among the 24 prospective studies, 6 studies [31,42,47,51,54,57] used longitudinal follow-up assessing chemotoxicity from baseline to after chemotherapy with multiple time points (at every cycle over 8 cycles [47,54]; at every 3 months over one year [51]; at every cycle over 12 cycles [31]; weekly over a month [42]; and monthly over 3 months [57]) over the course of chemotherapy. The most common time point to assess chemotherapy was a 6-month follow-up [7,35,39,49,50] throughout chemotherapy. None of the studies assessed chemotoxicity longer than a 1-year follow-up. Studies were conducted across various countries, predominantly in Europe (studies n = 15 [7,32,33,34,35,36,39,40,41,48,50,51,52,53,54,59]), followed by Asia (studies *n* = 6 [45,47,49,55,57,58]) and the United States (US) (studies *n* = 4 [37,38,43,44]). The sample sizes ranged from 2691 [40] to 52 [33], and the sample sizes were between ≥100 and <500 in the majority of studies (*n* = 16). Among the 30 studies, 24 studies [7,31,33,34,35,36,37,38,39,40,41,42,43,44,45,46,47,48,49,50,51,53,55,57] identified significant risk factors of chemotoxicity; 10 studies [31,33,37,39,40,43,46,48,49,50] examined the differences of chemotoxicity by categorical groups (i.e., high, versus low values) of risk factors; 11 studies reported odds ratios [ORs] [34,35,36,38,41,44,45,47,51,53,55,57]; and 2 studies [7,42] reported a Beta coefficient to present the associations of risk factors with chemotoxicity. Two studies found no significant risk factors for chemotoxicity [54,59], and four did not examine chemotoxicity risk factors [32,52,56,58]. 

**Table 1:** Characteristics of studies included for meta-analysis (N = 25).

Authors (Year)/ Location	Study Design	Cancer Types	Samples (N)/F(%)/ Mean Age [Range]	Primary Regimens	Prevalence of Top 2 Moderate-to-Severe Chemotoxicity Subgroups ^a^
Ali et al. (2016) [30]/ Canada and France	PL	CRC/ mixed stages	138/F50%/ 62 [28–87]	5-FU	GI (20%)/ Neuropathy (13%)
Antonio et al. (2018) [58]/ Europe	P	Older Adults CRC/ mixed stages	193/F32%/ 80 [75–89]	5-FU	Diarrhea (9.5%)/ Fatigue, Neutropenia (8.1%)
Aparicio et al. (2016) [31]/ Europe	P	Older Adults mCRC/IV	271/F42%/ 80 [75–92]	5-FU	Neutropenia (22%)/ GI (20.8%)
Backshall et al. (2011) [32]/ Europe	P	mCRC/IV	52/F35%/ 79 [42–86]	Cape	Hand-foot-syndrome (11%)/ Diarrhea (9%)
Barret et al. (2011) [33]/ Europe	P	mCRC/IV	114/F32%/ 65 [22–92]	5-FU	Neuropathy (85.5%)/ GI (28.7%)
Beukers et al. (2021) [34]/ Europe	R	Older Adults Colon/III	97/F52%/ 77 [70–85]	5-FU	Diarrhea (30%)/ Hand-foot syndrome (20%)
Breton et al. (2021) [35]/ Europe	P	mCRC/IV	2190/F38%/ 67 [59–75]	5-FU	Composite GI (15%)/ Neutropenia (13.2%)
Feliu et al. (2022) [7]/ Europe	P	Older Adults CRC/mixed stages	321/F32%/ 78 [70–90]	5-FU	Fatigue (12%)/ Diarrhea (10%)
Folprecht et al. (2008) [39]/ Europe	P	CRC/mixed stages	2691/F33%/ 70 [18–79]	5-FU	Neutropenia (28.9%)/ Diarrhea (20.5%)
Gallois et al. (2019) [40]/ Europe	P	Older Adults mCRC/IV	168/F44%/ 75 [70–92]	5-FU	Nausea, vomiting (15%)/ Diarrhea (8%)
Garg et al. (2012) [41]/ Australia	PL	CRC/III	173/F43%/ 63 [54–72]	5-FU	Neutropenia (55%)/ GI (50.6%, mucositis 12%)
Hochster et al. (2007) [43]/ USA	P	Older Adults CRC/ mixed stages	55/F47%/ 81 [75–90]	Leucovorin	GI (35.7%)/ Diarrhea (25%)
Jung et al. (2015) [44]/ S.Korea	P	Colon/III	229/F59%/ 61 [53–67]	5-FU	Neutropenia (40%)
Karabulut et al. (2022) [45]/Turkey	P	mCRC/IV	137/F61%/ 62 [18–83]	5-FU	Anemia (30%)/ GI (15.5%)
Li et al. (2021) [46]/China	PL	CRC/no data	233/F34%/ 58 [28–87]	Cape	Nausea (35%)/ Vomiting (35%)
Okada et al. (2017) [48]/ Japan	R	mCRC/IV	108/F56%/ 65 [34–83]	5-FU	Non-hematological (57%)/ Hematological toxicity (45%)
Osterlund et al. (2007) [49]/Europe	RCT	mCRC/IV	150/F49%/ 60 [31–75]	5-FU	GI (51%)/ Neutropenia (16%)
Retornaz et al. (2020) [50]/ Europe	PL	Older Adults Colon/ mixed stages	97/F51%/ 79 [70–90]	5-FU	Fatigue (64%)/ GI (40%)
Sastre et al. (2012) [51]/ Europe	P	Older Adults mCRC/IV	66/F42%/ 70 [70–86]	Cetuximab/ Cape	Neuropathy (16%)/ Diarrhea (16%)
Seymour et al. (2011) [52]/ Europe	P	mCRC/IV	440/F41%/ 74 [35–86]	5-FU	Pain (16%)/ Diarrhea (10%)
Stein et al. (2016) [53]/ Europe	PL	mCRC/IV	1249/F45%/ 74 [21–99]	Cape	Pain, Anemia, Diarrhea (12%)/ Nausea (8%)
Tominga et al. (2016) [54]/ Japan	R	CRC/III	135/F58%/ 63 [58–71]	5FU/Cape	Neutropenia (52%)/ Anorexia (17%)
Tsuchihashi et al. (2018) [55]/Japan	R	mCRC/IV	523/F41%/ 63 [55–85]	Regorafenib, Trifluridine, Tipiracil	Hand-foot-syndrome (20%)/ Anemia (12%)
Watanabe et al. (2018) [56]/Canada	PL	CRC/III	371/F49%/ 64 [60–89]	Cape/5-FU	GI (80%)/ Neuropathy (80%)
Yamada et al. (2013) [57]/ Japan	P	mCRC/IV	512/F36%/ 63 [33–79]	5-FU/OX	Neutropenia (43%)/ GI (12%)

Note: Cape: capecitabine; CRC: colorectal cancer; F: female; GI: gastrointestinal; 5-FU: 5-Fluorouracil; mCRC: metastatic CRC; and OX: oxaliplatin. Study designs: P: prospective study design; PL: prospective longitudinal design with repeated measures; R: retrospective design; and RCT: randomized controlled trials. ^a^ Toxicities measured by the Common Terminology Criteria for Adverse Events (CTCAE).

**Table 2:** Characteristics of studies not included for meta-analysis (N = 5).

Authors (Year)/ Location	Study Design	Cancer Stages	Samples(N)/ F(%)/ Mean Age [Range]	Primary Regimens	Chemotoxicity Measures	Prevalence of Top Moderate-to-Severe Chemotoxicity Subgroups
Brown et al. (2022) [36]/ USA	P	Colon/ II-III	533/ F56%/ 59 [47–70]	5-FU	Physicians’ chart and progress note review	Discontinuation of chemotherapy (13%)
Cespedes Feliciano et al. (2017) [37]/USA	P	Colon/ II-IV	533/ F55.4%/ 59 [no data]	5-FU	Physicians’ chart and progress note review and EMR ICD9 codes	Early discontinuation (36%)/ Neuropathy (24.1%)
Decoster et al. (2018) [38]/Europe	P	Older Adults mCRC/ IV	252/ F38%/ 77 [69–91]	5-FU	Physicians’ chart and progress note review	Vascular toxicity (35%)/ GI toxicity (13.6%)
Grimes, C. (2022) [42]/ USA	R	CRC/III	89/ F58%/ 62 [no data]	5-FU	Physicians’ chart and progress note review	Diarrhea (6.7%)/ Nausea (5.6%)
Looijaard et al. (2020) [47]/Europe	P	Older Adults Colon/III	53/ F45%/ 71 [68–74]	5-FU	Physicians’ chart and progress note review	Dose reduction/ incompletion (52.8%)

Note: CRC: colorectal cancer; EMR: electronic medical record; F: female; 5-FU: 5-Fluorouracil; GI: gastrointestinal; ICD-9: international classification of diseases, 9th Revision (ICD-9); mCRC: metastasis CRC; and PRO-CTCAE: patient-reported outcomes version of the common terminology criteria for adverse events.

### 3.3. Systematic Review: Prevalence and Risk Factors of Chemotoxicity

#### 3.3.1. Patient Characteristics

Among 12,706 samples across the 30 studies, most participants were male (56%). Stage IV was the most prevalent, representing an average of 59% of participants, followed by stage III (28% of total samples). The mean age across the 30 studies was 68.4 years old. Age ranged from 58 [47] to 81 years old [44] across the 30 studies. Ten studies [7,32,35,39,41,44,48,51,52,59] focused solely on older adults (≥65 years old) within the CRC population. The majority of participants were diagnosed with CRC, followed by colon cancer only (*n* = 6 [35,37,38,45,48,51]). Additionally, 14 studies were conducted on metastatic CRC (Table 1 and Table 2). 

#### 3.3.2. Prevalence of Clinician-Reported Chemotoxicity

The primary chemotherapy regimen in CRC was 5-FU-based agents (e.g., single use of 5-FU, FOLFIRI, FOLFOX, and capecitabine as an oral prodrug of 5-FU) [60] and the most common chemotoxicity measure was the Cancer Terminology Criteria for Adverse Events (CTCAE) grading scale, used in 25 studies among the 30 selected studies. Five studies [37,38,39,43,48] assessed the prevalence of chemotoxicity by reviewing electronic health records (EHRs), such as physicians’ charts and progress note reviews, which included the International Classification of Diseases (ICD)-9 codes (study *n* = 1 [38]). The majority of the studies reported GI toxicity as the most prevalent type of moderate-to-severe chemotoxicity, from 12% to as high as 80% in different studies, except for 6 [37,38,45,48,49,56] out of the 30 studies (Table 1 and Table 2). Diarrhea was a frequently reported symptom of GI toxicity in 11 studies [7,33,35,40,41,43,44,52,53,54,59], with prevalence rates ranging from 8% [41] to 30% [35] across the studies. The next most frequently reported top-ranked moderate-to-severe chemotoxicity across the studies was neutropenia in nine studies [32,36,40,42,45,50,55,57,59] with prevalence rates ranging from 8.1% [59] to 55% [42], followed by neuropathy in five studies [34,38,52,57], ranging from 13% [31] to 85.5% [34]. Three studies also evaluated chemotoxicity by examining the incidence of chemotherapy discontinuation or dose reduction/incompletion [37,38,48]. 

#### 3.3.3. Risk Factors of Clinician-Reported Chemotoxicity

Regarding individual risk factors of chemotoxicity (Table 3), four main categories were identified: nutritional status, geriatric assessment, biomarkers, and demographic/clinical factors. Fourteen studies [7,31,34,37,38,41,43,45,46,47,49,51,55,57] focused on malnutrition. The most common risk factor was low albumin levels [34,41,46,47,49,51,55]), followed by weight loss [7,34,41,46,47], low BMI [37,38,46], and low hemoglobin levels [47,57] before chemotherapy. Body composition, such as low muscle mass, sarcopenia, and high abdominal adiposity [37,38,43,45], were also significant factors. Body composition, including muscle and fat mass, were more sensitive risk factors of chemotoxicity than BMI, body weight, or body surface area. Seven studies [7,35,36,39,44,51,53] included a geriatric assessment, including a comprehensive frailty score, physical frailty–grip status, or performance levels, showing that the baseline frailty status is associated with chemotoxicity. Various biomarkers were studied with two major categories: (1) pro-inflammatory markers, e.g., white blood cell counts (WBCs) [7,47,53], C-reactive protein (CRP) [47,51,55], and lactate [40], and (2) hepato-renal functions [7,36,40,51]. Higher levels of low-density lipoprotein-derived lipids, short telomere length, lack of lactobacillus microbiome, and high carcinogenic antigen (CEA) levels were also shown to be associated with the risk of chemotoxicity. In demographic/clinical factors, the associations of age with chemotoxicity were inconsistent. Significant associations were found between a younger adult age group (<50 years old) and neutropenia [42,47], as well as GI toxicity [42]. On the other hand, older age (≥65 years old) was significantly associated with GI toxicity [44,47] and hematological toxicity [47,57]. Female sex, cancer stages, previous history of aggressive chemotherapy or colorectal surgeries, and baseline HRQoL were also significantly associated with chemotoxicity (Table 3). 

We also identified the lack of intervention to prevent and manage chemotoxicity as a potential risk factor for the incidence and severity of chemotoxicity among CRC patients. Only Osterlund et al. (2007) [50] conducted an RCT with *Lactobacillus* intervention in metastatic CRC. Compared to the no-intervention groups, grade 3 or 4 diarrhea incidence was lower in patients treated with *Lactobacillus* (22% vs. 37%, *p* = 0.027). Additionally, these patients experienced less abdominal discomfort, required fewer hospital visits, and had chemotherapy dose reductions due to GI toxicity.

### 3.4. Meta-Analysis: Prevalence and Risk Factors of Chemotoxicity

Among the 30 studies analyzed in this review, 25 were included for meta-analysis when considering consistent chemotoxicity measures (i.e., CTCAE) (Table 1).

#### 3.4.1. Publication Bias (Asymmetry and Heterogeneity) Assessment

**Figure 2:**
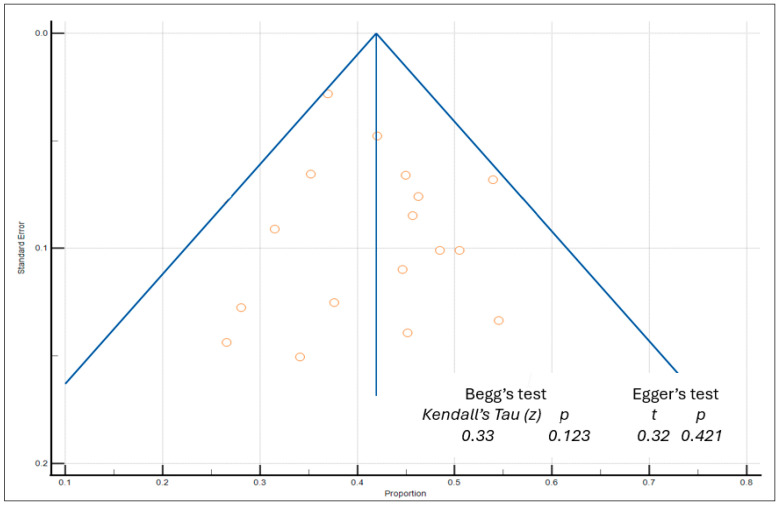
Funnel plot of publication bias (based on the 17 articles reporting prevalences of overall moderate-to-severe chemotoxicity data).

Among 25 studies in the meta-analysis, we performed a publication bias assessment, primarily based on the prevalence of overall moderate-to-severe chemotoxicity across 17 studies using the CTCAE grading scale (asymmetry shown in Figure 2; heterogeneity shown in Table 4). Then, we also performed a publication bias assessment based on the prevalence of chemotoxicity subgroups by rank, from the selected 25 studies (Table 5 and Supplementary Figure S1). No asymmetries from Begg’s and Egger’s tests and funnel plots were found in the 25 selected studies included for meta-analysis (Table 4 and Table 5, Figure 2, and Supplementary Figure S1). Overall, high levels of heterogeneity were observed in chemotoxicity prevalence and its subgroups, with I^2^ > 75%. Consequently, a random-effects model was utilized to account for heterogeneity among study results (Table 4 and Table 5).

#### 3.4.2. Pooled Prevalence of Chemotoxicity

**Figure 3:**
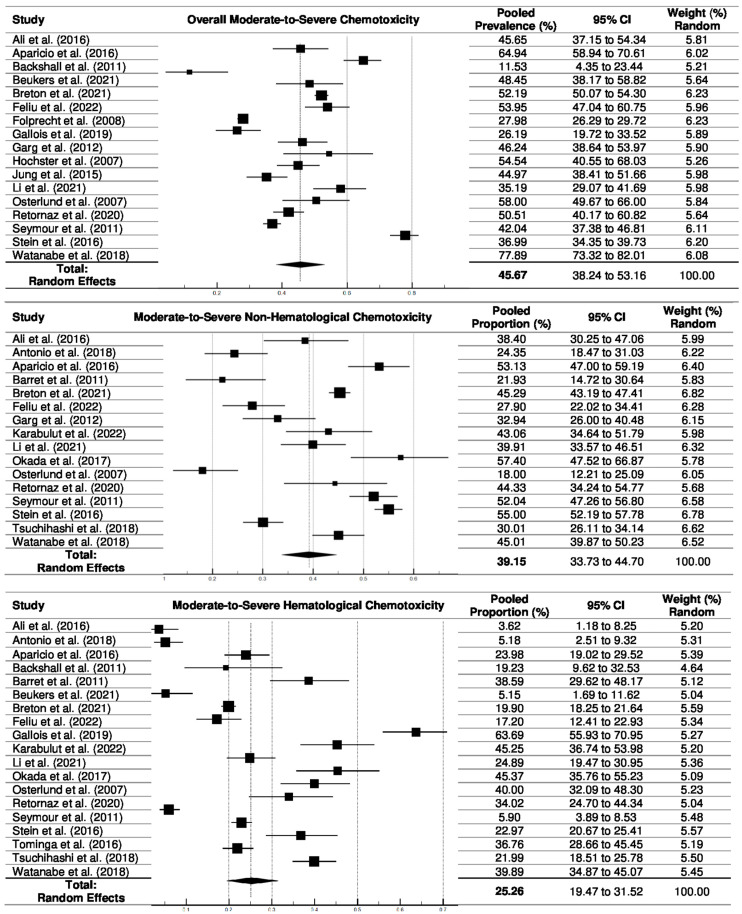
Forest plots for pooled prevalences of moderate-to-severe chemotoxicity (overall, non-hematological, and hematological toxicities) [7,30,31,32,33,34,35,39,40,41,43,44,45,46,47,48,49,50,52,53,54,55,56,58].

*Moderate-to-severe chemotoxicity*. The prevalence of moderate-to-severe overall chemotoxicity based on the CTCAE grading scale was reported in 17 studies [7,31,32,33,35,36,40,41,42,44,45,47,50,51,53,54,57]. The pooled prevalence of moderate-to-severe overall chemotoxicity was 45.7% (95% CI: 38.2 to 53.2) (Table 4 and Figure 3).

**Table 4:** Heterogeneity tests for publication bias among 17 studies included for meta-analysis of prevalence of overall chemotoxicity (moderate-to-severe).

Study Characteristics ^a^	Sub-Variables	Studies *n*; Total Sample *n*	Pooled Prevalence of Overall Chemotoxicity ^d^ % (95% CI)	Test for Heterogeneity between Studies ^e^		
				*Q_df_*_Between_	*I*^2^ (%)_Between_	*p*_Between_
**Total Studies ^a^**		17; *n* = 8819	45.7 (38.2 to 53.2)	112.54_16_	78.14	<0.001
**Location ^b^**	Europe	12; *n* = 7793	45.4 (36.3 to 55.3)	154.65_11_	98.32	<0.001
	Asia	2; *n* = 462	40.1 (30.8 to 49.8)	223.53_1_	99.41	0.049
**Publication year**	2007–2010	3; *n* = 2896	46.1 (23.6 to 29.7)	158.39_2_	95.49	0.012
	2011–2015	4; *n* = 894	37.0 (26.9 to 47.7)	133.41_3_	98.65	0.005
	2016–2020	6; *n* = 2294	55.9 (33.9 to 76.8)	148.85_5_	95.42	0.044
	2021–2024	4; *n* = 2735	47.5 (39.2 to 55.8)	133.52_3_	88.41	0.043
**Study design ^c^**	Observational	16; *n* = 8669	45.5 (37.1 to 53.5)	140.89_15_	96.89	0.023
**Female prevalence**	<50.0%	13; *n* = 8258	47.6 (37.6 to 57.7)	35.32_12_	91.23	0.010
	≥50.0%	4; *n* = 561	46.7 (42.6 to 50.8)	28.52_3_	89.41	0.043
**Sample size**	*n* < 100	4; *n* = 301	40.5 (22.9 to 59.4)	158.42_3_	78.75	0.044
	100 ≤ *n* < 500	10; *n* = 2388	52.9 (39.6 to 65.9)	192.52_9_	66.49	0.013
	*n* ≥ 500	3; *n* = 6130	38.8 (24.3 to 54.5)	151.45_2_	74.32	0.007
**Age group** **(Participants’ age range)**	18 ≤ age < 65	11; *n* = 7916	33.4 (22.3 to 39.5)	185.89_10_	85.65	0.041
	65≥ Older Adults	6; *n* = 903	50.1 (27.6 to 69.9)	133.55_5_	86.56	0.035
**Cancer Types**	Colon only	4; *n* = 870	47.1 (42.3 to 51.8)	321.32_3_	98.61	0.019
	Mixed stages CRC	5; *n* = 3196	48.8 (32.0 to 65.7)	139.32_4_	88.53	0.043
	Metastatic CRC	7; *n* = 4520	41.9 (32.9 to 51.4)	98.49_6_	89.65	0.041

Note: CIs: confidence intervals; CRC: colorectal cancer; df: degree of freedom; and GI: gastrointestinal. ^a^ We included only 17 studies reporting overall chemotoxicity prevalence with moderate-to-severe severity using the Common Terminology Criteria for Adverse Events (CTCAE) measure among 25 studies included in the meta-analysis in our study. ^b^ We excluded Ali et al. [30], Hochster et al. [43], and Watanabe et al. [56], as these studies are the sole representation of a particular country among a total of 25 selected studies of this study. ^c^ We excluded Osterlund et al. (2007) [49] from the RCT study. ^d^ Moderate-to-severe toxicity: weighted effect size and standard error were applied, resulting in pooled prevalence and 95% confidence intervals (CIs). ^e^ *p*-value < 0.05 is considered significant for heterogeneity tests.

We also performed subgroup analyses of the pooled prevalence of moderate-to-severe chemotoxicity by stratifying prevalence according to subgroups of chemotoxicity (e.g., non-hematological, hematological, and others) (Table 5, and funnel plots in Supplementary Figure S2). In moderate-to-severe chemotoxicity subgroups, the pooled prevalences of moderate-to-severe non-hematological and hematological toxicities was 39.2% (33.7 to 44.7) and 25.3% (19.4 to 31.5), respectively. Among moderate-to-severe other chemotoxicity subgroups, abdominal pain was the most frequent (24.3%), followed by GI toxicity (22.9%), neuropathy (17.9%), neutropenia (17.8%), nausea/vomiting (17.8%), and diarrhea (14.1%) (Table 4 and Supplementary Figure S2).

**Figure 4:**
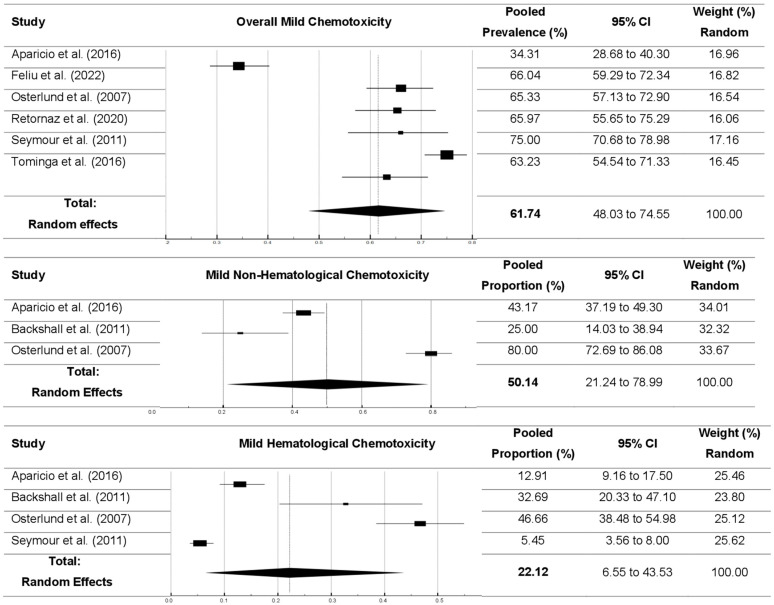
Forest plots for pooled prevalences of mild chemotoxicity (overall, non-hematological, and hematological toxicities) [7,31,32,49,50,52,54].

*Mild chemotoxicity*. Only a few studies reported the prevalence of chemotoxicity with mild severity. Six studies [7,32,50,51,53,55] were included for the pooled prevalence of mild overall chemotoxicity, resulting in a 61.7% pooled prevalence. The pooled prevalences of mild non-hematological and hematological toxicities was 50.1% and 22.5%, respectively (Figure 4 and Table 5). Among chemotoxicity subgroups with mild severity (Table 5 and Supplementary Figure S3), diarrhea was the most frequent chemotoxicity (58.9%), followed by hand–foot syndrome (51.7%), anemia (36.5%), and mucositis/stomatitis (28.6%).

**Table 5:** Asymmetry and heterogeneity tests for publication bias: subgroups of chemotoxicity.

Study Sub-Variables	Studies *n*; Total Sample *n*	Pooled Prevalence by Rank (%: 95% CI) ^a^	Asymmetry Tests				Heterogeneity Tests between Studies		
			Begg’s Test		Egger’s Test				
			Kendall’s *T*	*p* ^b^	*SE*	*p* ^c^	*Q_df_* _Between_	*I*^2^ (%)_Between_	*p* ^b^_Between_
**Pooled Prevalence of Moderate-to-Severe Chemotoxicity**									
Non-Hematological Toxicity	16; *n* = 6602	39.2 (33.7 to 44.7)	0.34	0.600	40.9	0.943	121.69_15_	88.56	0.033
Hematological Toxicity	19; *n* = 6882	25.3 (19.4 to 31.5)	0.29	0.491	20.5	0.423	132.65_18_	93.79	0.049
**Pooled Prevalence of Moderate-to-Severe Chemotoxicity Subgroups**									
Abdominal pain	3; *n* = 1655	24.3 (2.01 to 60.1)	0.33	0.412	60.3	0.143	56.1_2_	99.61	<0.001
GI Toxicity	20; *n* = 9489	22.9 (16.4 to 30.1)	0.23	0.598	20.40.	0.892	132.52_19_	98.59	<0.001
Neuropathy	9; *n* = 5382	17.9 (4.9 to 36.5)	0.4/2	0.394	30.2	0.352	122.59_8_	95.89	0.049
Neutropenia	14; *n* = 8227	17.9 (11.1 to 25.9)	0.51	0.341	50.3	0.422	144.65_13_	98.51	<0.001
Nausea/Vomiting	10; *n* = 5089	17.8 (8.5 to 29.6)	0.41	0.535	50.6	0.314	69.5_9_	98.66	<0.001
Diarrhea	18; *n* = 7209	14.1 (11.1 to 17.4)	0.31	0.591	40.4	0.289	144.01_17_	98.11	<0.001
Leukocytosis	2; *n* = 2287	12.9 (0.4 to 38.2)	0.32	0.542	30.4	0.132	243.31_1_	99.51	0.035
Fatigue	7; *n* = 2772	12.6 (4.6 to 23.7)	0.38	0.224	40.5	0.499	132.56_6_	99.92	<0.001
Anemia	7; *n* = 2547	10.4 (4.5 to 18.3)	0.56	0.621	30.4	0.289	132.55_6_	89.61	<0.001
Leukopenia	3; *n* = 4452	10.2 (3.6 to 19.6)	0.41	0.214	50.6	0.512	241.95_2_	89.52	0.021
Mucositis/stomatitis	7; *n* = 2608	9.5 (2.9 to 18.7)	0.59	0.399	60.2	0.526	87.4_6_	95.41	0.014
Hand-Foot-Syndrome	5; *n* = 700	9.0 (1.8 to 20.9)	0.16	0.841	60.5	0.431	353.17_4_	97.84	0.019
Anorexia	5; *n* = 1804	6.4 (3.7 to 9.6)	0.41	0.412	50.4	0.122	101.42_4_	99.41	<0.001
Coagulation disorders	2; *n* = 2757	4.9 (4.1 to 5.8)	0.33	0.312	50.5	0.082	99.49_1_	89.59	<0.001
Constipation	2; *n* = 341	3.4 (1.8 to 5.6)	0.14	0.623	50.6	0.412	93.04_1_	84.23	0.048
**Pooled Prevalence of Mild Chemotoxicity**									
Overall Chemotoxicity	6; *n* = 1309	61.7 (48.0 to 74.6)	0.54	0.841	20.3	0.347	122.68_5_	95.92	<0.001
Non-Hematological Toxicity	3; *n* = 473	50.1(21.1 to 78.9)	0.55	0.792	30.1	0.572	134.88_2_	92.51	0.002
Hematological Toxicity	4; *n* = 913	22.1 (6.6 to 43.5)	0.41	0.852	20.9	0.123	156.41_3_	85.91	0.024
**Pooled Prevalence of Mild Chemotoxity Subgroups**									
Diarrhea	4; *n* = 956	58.9 (28.8 to 85.6)	0.41	0.312	20.4	0.312	111.31_3_	78.41	0.048
Hand-Foot-Syndrome	2; *n* = 202	51.7 (0.4 to 98.8)	0.52	0.411	40.1	0.522	198.56_1_	88.51	0.009
Anemia	3; *n* = 763	36.5 (1.5 to 84.9)	0.33	0.012	20.3	0.731	153.66_2_	94.55	0.014
Mucositis/stomatitis	3; *n* = 884	28.6 (7.2 to 57.0)	0.28	0.102	40.9	0.341	142.65_2_	93.41	<0.001
Neutropenia	3; *n* = 884	25.7 (6.2 to 52.3)	0.62	0.531	50.3	0.512	166.24_2_	99.79	<0.001
Nausea/Vomiting	3; *n* = 1572	18.3 (0.6 to 52.3)	0.38	0.512	20.5	0.823	143.66_2_	95.12	<0.001
Fatigue	2; *n* = 492	17.1 (0.5 to 63.7)	0.12	0.312	20.4	0.623	98.42_1_	98.55	0.032
Coagulation disorders	2; *n* = 1422	4.3 (0.7 to 10.7)	0.52	0.432	20.3	0.412	102.03_1_	85.66	0.045

Note: CIs: confidence intervals; GI: gastrointestinal; df = degree of freedom; and SE: standard error. ^a^ Weighted effect size and standard error were applied, resulting in pooled estimates and 95% confidence intervals (CIs). ^b^ *p* < 0.05 considered significant publication bias. ^c^ *p*-value of <0.05 was considered statistically significant.

#### 3.4.3. Pooled Associations of Age as a Predictor of Chemotoxicity Prevalence

In our review of significant risk factors for chemotoxicity (Table 3), we identified multiple inconsistencies. These inconsistencies were present in the types of statistical correlations used (e.g., ORs, Beta coefficients, or group comparisons) and in chemotoxicity subgroups (e.g., prevalences of overall toxicity, hematological, non-hematological, GI toxicities, or neutropenia). Given these inconsistencies, age was the only risk factor available for computing pooled associations with the prevalence of chemotoxicity (Table 6 with age as a continuous variable, and 7 with age as a group variable). In unadjusted meta-regression models, there were significant relationships between age (continuous variables) and chemotoxicity (Table 6). When all other variables held constant, an increase of 1 year in age was negatively associated with an average 1.09 change (unstandardized B = −1.09) in the prevalence of moderate-to-severe neutropenia (*p* = 0.003), while age was positively associated with an average change in B units in the prevalence of nausea/vomiting (B = 1.32, *p* = 0.028), diarrhea (B = 1.05, *p* = 0.012), and GI toxicity (B = 1.02, *p* = 0.044). Given the standardized β coefficients in unadjusted models, age was the most significantly associated with GI toxicity (β = 1.97, *p* = 0.005), followed by the prevalence of neutropenia (β = −1.78, *p* = 0.002). In adjusted meta-regression models (controlling for cancer stage as the most prevalent cancer stage, metastatic status, cancer type, and sample size as group variables, and sex as the most prevalent sex group variable) (Table 6) similar results were found, showing a negative association between age and neutropenia, while positive associations were found between age and nausea/vomiting, diarrhea, and GI toxicity prevalences. Given the standardized β coefficients in the adjusted models (Table 6), age was the most significantly associated with the prevalence of GI toxicity (β = 1.85, *p* = 0.001), followed by the prevalence of neutropenia (β = −1.44, *p* = 0.004). No significant relationship existed between age and overall, non-hematological, and hematological chemotoxicities in the unadjusted and adjusted models (Table 6).

Table 7 presents the results of a meta-ANOVA (F tests) and regressions (ORs) comparing the prevalence of different types of chemotoxicity between two age groups: adults (age ≥ 18 and <65 years old) and older adults (age ≥ 65 years old). Significant differences existed in the prevalence of overall chemotoxicity, neutropenia, diarrhea, and GI toxicity between adults and older adults. The mean prevalences of overall chemotoxicity, diarrhea, and GI toxicity were higher in the older adult group compared to the adult group, while this was lower in the older adult group compared to the adult group for neutropenia. Compared to adult groups, older adult groups reported higher prevalences of chemotoxicity, with 14% in overall chemotoxicity (OR = 1.14), 27% in diarrhea (OR = 1.27), and 65% in GI toxicity (OR = 1.65). However, they reported a 35% lower prevalence of chemotoxicity in neutropenia (OR = 0.65).

## 4. Discussion

This study is the first to systematically review clinician-reported chemotoxicity and perform a meta-analysis to compute the pooled prevalence of chemotoxicity and the pooled associations of risk factors of chemotoxicity in CRC patients. Our study has added new information to the comprehensive understanding of the current literature on chemotoxicity in CRC populations. It provides insights into the pooled prevalence and risk factors of clinician-reported chemotoxicity, including its subgroups, to foster a better consensus on this topic. We showed evidence to suggest a significant prevalence of chemotoxicity and several risk factors of chemotoxicity in CRC patients. New findings gathered from the current study include the overall prevalence of chemotoxicity (45.7% for moderate-to-severe levels and 61.7% for mild severity levels), indicating that the problem of chemotoxicity needs to be improved in CRC patients. Our subgroup analyses also provided further details of the prevalences of subgroups of chemotoxicity, showing that non-hematological toxicity was more common compared to hematological toxicity regardless of the severity.

Among subgroups of chemotoxicity, GI-related side effects were prevalent regardless of the severity (e.g., moderate-to-severe toxicity: abdominal pain, composite GI toxicity, and nausea/vomiting, diarrhea; mild toxicity: diarrhea, mucositis/stomatitis, and nausea/vomiting). This finding was corroborated by the systematic review and meta-analysis of patient-reported outcomes (PROs) in CRC patients, which showed prevalent and severe GI symptoms (mean prevalences 40%) after cancer treatments [5], compared to other cancers (mean GI toxicity prevalence < 5% in breast cancer [61] and <10% in lung cancer [62]). However, fatigue was less prevalent (12.6% for moderate-to-severe fatigue and 17.1% for mild fatigue), and no psychological distress data were reported by clinicians in our review. Conversely, previous literature [5] reported that fatigue and psychological distress were the most severe and prevalent among CRC patients. The discrepancy between patient-reported toxicities and those assessed by clinicians using the CTCAE can be attributed to several factors. Evidence suggests that clinician-reported adverse events may miss or under-report up to 50% of symptoms that patients experience during treatment [63]. Clinicians might not fully recognize or document all treatment-related adverse events, especially more subjective ones like fatigue, depression, and sleep disturbances [63]. In conclusion, both PROs and clinician-reported chemotoxicity provide valuable and complementary insights. Including both data types in chemotoxicity assessment and management appears to be warranted.

Our study suggests that chemotoxicity assessment and management should consider various subgroups (e.g., non-hematological or hematological) due to differing etiologies [64]. Inconsistencies in risk factor measures and chemotoxicity outcome subgroups (i.e., hematological, GI toxicity, neuropathy, etc.), along with statistical methods, limited our meta-regressions. Despite this, our findings on common chemotoxicity risk factors will aid in identifying high-risk groups and the future development of prediction models or targeted interventions. In our findings, individual components were identified as frequent chemotoxicity risk factors, including malnutrition, frailty, hepato-renal functions, pro-inflammatory status (increased WBC or CRP), previous colorectal surgical histories, cancer stages, chemotherapy doses, and baseline HRQoL. Among interventional components to prevent and manage chemotoxicity, only *Lactobacillus* (diet) RCT improved chemotoxicity [50]. Furthermore, our systematic review (Table 2) and meta-analyses (Table 5) investigating the common risk factors of chemotoxicity showed consistent results for age: age was positively associated with composite and individual GI toxicities but negatively with neutropenia. This was consistent with a previous study on 927 older adult patients with mixed cancer types (breast 33%, lung 19%, and colon 10%) [64]. A potential explanation is that an age-related decline in GI function (i.e., leaky gut, slow transition, and dysbiosis) can exacerbate the vulnerability of mucosal damage in the gut triggered by CRC pathology per se and chemotherapy [65]. Additionally, certain drugs, such as 5-FU (the primary treatment agent for CRC) or immune checkpoint inhibitors (for metastatic CRC), can have more severe side effects on the GI tract in CRC patients [66]. Our negative associations could be due to the use of lower doses or less aggressive chemotherapy regimens in older adults due to concerns about treatment tolerance and comorbidities [64,67].

In our review, malnutrition status was measured using weight loss, BMI, albumin or hemoglobin levels, and muscle or fat mass. Emphasis was placed on muscle or fat mass as more sensitive chemotoxicity indicators than body weight or BMI. Previous reviews in nutritional status and chemotherapy tolerance (e.g., chemotoxicity, treatment non-compliance, dose reduction, etc.) in CRC [33,34] corroborate our findings, suggesting an association between lean muscle mass and chemotoxicity and the importance of nutritional screening before initiating chemotherapy. This could be because a low lean mass might be associated with frailty, greater comorbidities, and slow drug metabolisms of hydrophilic chemotherapy (such as 5-FU), leading to more incidences of chemotoxicity [40]. On the other hand, a high fat mass could be involved in poor drug distribution and clearance, impacting the increased degree of toxicity [40]. However, the potential mechanisms for the association between body composition and chemotoxicities remain unclear. Our review identified frailty (performance, cognition, or comprehensive frailty scores) as a predictor of chemotoxicity. This is consistent with a previous review study [5], which found that frailty predicted HRQoL and symptom toxicity. Physical frailty and malnutrition statuses, such as sarcopenia, were closely related. Therefore, further monitoring of chemotoxicity for patients with both frail and malnourished conditions is warranted, such as dietary evaluations by a nutritionist.

Increased WBC and CRP significantly predicted chemotoxicity in our study. This could be due to a high tumor burden and altered immune responses [68]: high WBC and CRP levels can also indicate a larger tumor burden. Larger tumors may require more aggressive chemotherapy, leading to increased chemotoxicity. Furthermore, both high WBC and CRP levels indicate an activated immune response. Chemotherapeutic agents can cause damage to the immune system, particularly to T cells, and an already activated pro-inflammatory status may exacerbate this damage, leading to worse chemotoxicity triggered by altered immune and inflammatory functions [68].

Anti-inflammatory interventions, such as lifestyle or microbiome modifications, demonstrated anti-inflammatory effects in cancer patients [69]. Lactobacillus microbiome intervention was the only intervention study in CRC patients to examine the chemotoxicity identified in our study [50]. The microbiome has been associated with multiple symptoms, including GI, psychological distress, dermatological disorders, neuropathy, and hematological complications in many chronic diseases [69]. Therefore, anti-inflammatory or microbiome-targeted interventions can be considered for pre- and post-rehabilitation for chemotherapy for this patient group. Despite several intervention studies, including a geriatric assessment [70], muscle resistance exercise [71], and nutritional [72] interventions aiming to reduce chemotoxicity, none of these studies focused primarily on CRC [70,72], and examined clinician-reported chemotoxicity [71,72]. Our review of risk factors of chemotoxicity also revealed limited interventions for preventing and managing chemotoxicity in CRC. Consistently, none of the 30 analyzed studies conducted interventions such as predicting and screening for chemotoxicity or implementing physical and psychological interventions to manage chemotoxicity.

Clinical Implications. Our study underscores the need for targeted approaches to prevent and manage chemotoxicity in CRC patients undergoing chemotherapy. Consideration of specific subgroups of chemotoxicity, such as GI toxicity, neuropathy, and neutropenia, is crucial for tailoring interventions effectively. Oncology professionals should explore diverse strategies, such as nutritional, anti-inflammatory, and pain interventions, to address different subgroups of chemotoxicity. Pre-screening for potential risk factors, like age, nutritional status, and frailty, is essential to anticipate and mitigate adverse effects. Rather than relying solely on chronological age, a comprehensive assessment of multiple risk factors (e.g., age–nutritional status–chemotherapy doses and durations) is recommended to guide treatment decisions. Balancing treatment benefits with side effect risks is vital for older patients who may be more vulnerable. Conversely, younger patients may face increased chemotoxicity risks due to aggressive treatment regimens. Early discussions regarding individualized goals of care are paramount for minimizing chemotoxicity and optimizing treatment outcomes.

Future Research. Future research should consider precision medicine approaches to tailor individualized supports mitigating chemotoxicity based on patient characteristics, aiming to minimize chemotoxicity. To achieve this, the primary steps of research should include the following: exploring the underlying socio-biological mechanisms of chemotoxicity, including its subgroups; developing chemotoxicity prediction models by considering the most significant risk factors and integrating biomarkers involved in immune and inflammatory systems (e.g., hematological immune parameters, microbiome or metabolomics) to identify at-risk groups; and designing and testing personalized interventions that apply various strategies (e.g., nutrition, anti-inflammatory, social support-focused, or health system changes), primarily targeting common risk factors of overall chemotoxicity, or specific to chemotoxicity subgroups in CRC, and integrating patient preferences into treatment decisions.

Limitations. Although the overall meta-analysis yielded significant findings, several subgroups of chemotoxicity (e.g., coagulation disorders and constipation) were reported in a small number of included studies. The endpoint of measuring chemotoxicity over the course of chemotherapy varied by study. Thus, longitudinal trajectories of chemotoxicity are unclear. We only conducted meta-regressions with age variables due to inconsistent measures and statistical methods across studies in other risk factors of chemotoxicity. Other important risk factors could require further investigation of their relationships with chemotoxicity. For example, other potential predictors (e.g., which types of chemotherapy regimens are the most significant risk factors for each subgroup of chemotoxicity prevalence?) remain unknown in our study. There may be uncontrolled potential covariate factors (e.g., years since cancer diagnosis, types, doses, duration of chemotherapy regimens, and comorbidities), limiting the generalizability of our findings. Lastly, in our study, the random-effects model gives more weight to studies with larger sample sizes, which can lead to biased estimates of our meta-analytic results [29].

## 5. Conclusions

This study reveals chemotherapy’s impact on chemotoxicity, especially non-hematological and GI toxicity. It highlights the interplay between various factors and chemotoxicity, emphasizing the necessity for personalized strategies considering chemotoxicity risk factors such as malnutrition, frailty, certain immune and inflammatory biomarkers, and patient characteristics like age. Understanding these factors is crucial in predicting the potential harm of chemotoxicity in patients with CRC. While PRO assessments are important, clinician-reported toxicity by considering objective factors can assist in better prevention and management of chemotoxicity. Ongoing research exploring the underlying mechanisms of chemotoxicity, including its subgroups and patient-centered prediction and intervention models, is crucial for optimizing patient well-being and treatment success.

## Figures and Tables

**Table 3:** Significant individual risk factors ^a^ of overall chemotoxicity (moderate-to-severe).

Authors (Year) ^b^/ Statistical Methods	Nutrition	Geriatric Assessments	Biomarkers	Demographic/ Clinical Factors
Ali et al. (2016) [30]/ Group comparisons	Body mass with neuropathy			
Backshall et al. (2011) [32]/ Group comparisons			Metabolic lipid panel	
Barret et al. (2011) [33]/ Odds Ratios (ORs)	Weight loss, low albumin			
Beukers et al. (2021) [34]/ ORs		Comprehensive Frailty		Female sex/ Cancer stages
Breton et al. (2021) [35]/ ORs		Physical Frailty (Performance)	Alkaline phosphatase (ALP)	Surgical history. Hx of aggressive chemotherapy
Feliu et al. (2022) [7]/ Beta coefficient	Weight loss	Comprehensive Frailty	Kidney function	
Folprecht et al. (2008) [39]/ Group comparisons			High WBC, ALP, lactate	
Gallois et al. (2019) [40]/ ORs	Weight loss, low albumin			
Garg et al. (2012) [41]/ Beta Coefficient			Short telomere length, high platelet lymphocyte ratio, and low neutrophil count with hematological and GI toxicity	Younger age with neutropenia and GI toxicity
Hochster et al. (2007) [43]/ ORs		Physical Frailty (Performance)	CEA, liver panels, creatine	Older age with GI toxicity (diarrhea)
Jung et al. (2015) [44]/ ORs	Psoas muscle mass			
Karabulut et al. (2022) [45]/ Group comparisons	Low BMI, Weight loss, low albumin			
Li et al. (2021) [46]/ ORs	Weight loss with hand–foot syndrome, and nausea, low hemoglobin and albumin with hematological toxicity.		Increased WBC, high CRP with hematological toxicity	Older age with GI and hematological toxicity. Younger age with neutropenia.
Okada et al. (2017) [48]/ Group comparisons	Low albumin with hepatotoxicity			
Osterlund et al. (2007) [49]/ Group comparisons			Lactobacillus	
Retornaz et al. (2020) [50]/ ORs	Low albumin	Physical Frailty (Grip strength, Performance)	Increased CRP, and ALP	Hx of aggressive chemotherapy
Seymour et al. (2011) [52]/ ORs		Physical Frailty (Performance)	Increased WBC	Baseline quality of life
Tominga et al. (2016) [54]/ ORs	Low albumin		CRP/albumin ratio. Elevated neutrophil/lymphocyte ratio	
Watanabe et al. (2018) [56]/ ORs	Low hemoglobin with hematological toxicity			Increased age and female sex with hematological toxicity
Brown et al. (2022) [36] */ Group comparisons	BMI and abdominal adiposity			
Cespedes Feliciano et al. (2017) [37] */ORs	BMI, muscle mass index			
Decoster et al. (2018) [38] */ Group comparisons		Physical Frailty (Performance)		
Grimes, C. (2022) [42] */ Group comparisons	Sarcopenia			
Looijaard et al. (2020) [47] */ Group comparisons				Hx of aggressive chemotherapy

Note. Alb: albumin; BMI: body mass index; CEA: carcinoembryonic antigen; CRP: C-reactive protein; GI: gastrointestinal; Hx: history; ORs: odd ratios; USA: United States of America; and WBC: white blood cell count. ^a^ Associated with multiple types of chemotoxicity unless otherwise specified. ^b^ We excluded studies with no significant risk factors (Antonio et al. 2018 [58]; Stein et al. 2016 [53]), or without examining risk factors of chemotoxicity (Aparicio et al. 2016 [31]; Sastre et al. 2012 [51]; Tsuchihashi et al. 2018 [55]; Yamada et al. 2013 [57]). * Studies not included in the meta-analysis (*n* = 5) due to unavailable chemotoxicity prevalence data or data that were not comparable for inclusion in the meta-analysis.

**Table 6:** Meta-Analyses (Age as a continuous variable): relationships of age with prevalence of chemotoxicity (moderate-to-severe levels).

Meta-Regression ^a^: Age (Continuous Variable) and the Prevalence of Chemotoxicity													
	Unadjusted Analyses						Adjusted Analyses ^b^						
Age (y.o.): Predictor	Unstandardized ^c^ B			Standardized ^c^ β			Unstandardized ^c^ B			Standardized ^c^ β			Sample *n*
	B (SE)	t	*p* ^d^	β (SE)	t	*p* ^d^	B (SE)	t	*p* ^d^	β (SE)	t	*p* ^d^	
Overall chemotoxicity	0.54 (0.49)	0.03	0.512	0.62 (0.25)	0.47	0.640	0.44 (0.32)	0.01	0.211	0.56 (0.33)	0.35	0.879	8819
Non-Hematological chemotoxicity	0.40 (0.40)	0.11	0.413	0.89 (0.97)	0.35	0.396	0.35 (0.29)	0.21	0.314	0.93 (0.34)	0.12	0.145	6602
Hematological chemotoxicity	0.41 (0.38)	0.09	0.331	0.76 (0.51)	0.15	0.574	0.51 (0.43)	0.04	0.421	1.21 (0.45)	0.41	0.652	6882
Neutropenia	−1.09 (0.56)	2.13	**0.003**	−1.78 (0.54)	2.41	**0.002**	−1.01 (0.41)	3.12	**0.003**	−1.44 (0.39)	1.98	**0.004**	8227
Nausea/ Vomiting	1.32 (0.85)	3.13	**0.028**	1.72 (0.56)	3.35	**0.001**	1.14 (0.72)	2.19	**0.012**	1.65 (0.56)	3.12	**0.001**	5089
Diarrhea	1.05 (0.31)	2.41	**0.012**	1.51 (0.29)	3.16	**0.004**	1.01 (0.31)	2.41	**0.001**	1.33 (0.32)	2.91	**0.005**	7209
GI toxicity	1.02 (0.44)	3.01	**0.044**	1.97 (0.43)	2.11	**0.005**	1.03 (0.35)	2.98	**<0.001**	1.85 (0.32)	2.53	**0.001**	9489

Note: Age: at the time of surveys; GI: gastrointestinal; SE: standard error. ^a^ Weighted least squares were applied (i.e., sample size weight adjustment). Types of chemotoxicity were selected only if the number of studies measuring the chemotoxicity was ≥10. ^b^ Adjusted regression models included prevalent cancer stages, metastatic status, cancer types, primary types of chemotherapy regimens, sample size (group categories), and sex group variables (female versus male prevalent). ^c^ Unstandardized B coefficient is interpreted as all other variables held constant; an increase of 1 year in age is associated with an average change in Beta units in the prevalence of chemotoxicity (moderate-to-severe). The standardized β (Beta) coefficient is interpreted as follows: for every one standard deviation increase in age, the outcome variables are expected to increase by β standard deviations, holding other variables constant. ^d^ *p*-value of <0.05 was considered statistically significant. Significant findings (*p* < 0.05) are highlighted in bold.

**Table 7:** Meta-Analyses (Age as a group variable): relationships of age with prevalence of chemotoxicity (moderate-to-severe levels).

Age Group and the Prevalence of Chemotoxicity (Meta-ANOVA and Meta-Regression) ^a^						
	Meta-ANOVA					Meta-Regression
	Adult Group (All Participants’ Age Range from ≥18 to <65 Years Old)		Older Adult Group (All Participants’ Age ≥65 Years Old)		F/*p* ^c^	Adjusted OR ^b,c^ (95% CIs) Ref. Adult Group
	Mean (SD)	Sample *n*	Mean (SD)	Sample *n*		
Overall Chemotoxicity	43.5(5.1)	7916	49.8 (5.3)	903	4.898, **0.002**	1.14 (1.01, 1.56), ***p* = 0.045**
Non-Hematological chemotoxicity	39.6 (3.8)	5476	37.4(6.8)	1126	0.214, 0.643	0.84 (0.75, 1.32), *p* = 0.984
Hematological chemotoxicity	24.3 (4.4)	5483	22.0 (8.4)	1399	0.106, 0.745	1.35 (0.85, 1.56), *p* = 0.845
Neutropenia	24.3 (6.9)	7396	13.7 (4.7)	831	5.382, **0.002**	0.65 (0.32, 0.95), ***p* = 0.031**
Nausea/Vomiting	22.9 (5.2)	4498	18.3 (7.6)	591	0.045, 0.831	0.98 (0.75, 1.32), *p* = 0.652
Diarrhea	14.2 (2.9)	6007	16.8 (4.3)	1202	3.214, **0.003**	1.27 (1.05, 1.95), ***p* = 0.003**
GI toxicity	18.1 (4.2)	8472	25.9 (4.8)	1017	2.494, **<0.001**	1.65 (1.12, 2.01), ***p* = 0.026**

Note: Age: at the time of surveys; CIs: confidence intervals; GI: gastrointestinal; OR: odds ratio; SD: standard deviation; ^a^ Weighted least squares were applied (i.e., sample size weight adjustment). Types of chemotoxicity were selected only if the number of studies measuring the chemotoxicity was ≥10. ^b^ Adjusted regression models included prevalent cancer stages, metastatic status, cancer types, primary types of chemotherapy regimens, sample size (group categories), and sex group variables (female versus male prevalent). ^c^ *p*-value of <0.05 was considered statistically significant. Significant findings (*p* < 0.05) are highlighted in bold.

## Data Availability

The authors affirm that this systematic review and meta-analysis has been reported in line with the PRISMA guidelines. The extracted data from the published papers supporting this study’s findings and review protocol are available from the corresponding author upon reasonable request.

## Supplementary Materials

The following supporting information can be downloaded at: https://www.mdpi.com/article/10.3390/cancers16142597/s1. Table S1: PRISMA 2020 Checklist; Table S2: Search Strategies; Table S3: Quality Assessment by Critical Appraisal Skills Program (CASP) Checklist; Table S4: Detailed Characteristics of Studies included for Systematic Review and Meta-Analysis; Figure S1: Funnel Plots for Asymmetry Tests: Subgroups of Prevalences of Chemotoxicities; Figure S2: Forest Plots for Pooled Prevalences of Chemotoxicities (Subgroups with Moderate-to-Severe Severity); Figure S3: Forest Plots for Pooled Prevalence of Chemotoxicities (Subgroups with Mild Severity).
