# Supplementary material for: Chemotoxicity and Associated Risk Factors in Colorectal Cancer: A Systematic Review and Meta-Analysis
Source: Cancers (Basel). 2024 Jul 20;16(14):2597. doi: 10.3390/cancers16142597 (PMC11274507; doi:10.3390/cancers16142597)

Supplementary Materials

Supplementary Tables

1. PRISMA 2020 Checklist
2. Search Strategies
3. Quality Assessment by Critical Appraisal Skills Program (CASP) Checklist
4. Detailed Characteristics of Studies included for Systematic Review and Meta-Analysis

Supplementary Figures

1. Funnel Plots for Asymmetry Tests: Subgroups of Prevalences of Chemotoxicities
2. Forest Plots for Pooled Prevalences of Chemotoxicities (Subgroups with Moderate-to-Severe Severity).
3. Forest Plots for Pooled Prevalence of Chemotoxicities (Subgroups with Mild Severity)

Supplementary Table S1. PRISMA 2020 Checklist

Section and Topic	Item #	Checklist item	Location where item is reported
**TITLE**			
Title	1	Identify the report as a systematic review.	Front page
**ABSTRACT**			
Abstract	2	See the PRISMA 2020 for Abstracts checklist.	Page 2.
**INTRODUCTION**			
Rationale	3	Describe the rationale for the review in the context of existing knowledge.	Page 3, lines 118-155.
Objectives	4	Provide an explicit statement of the objective(s) or question(s) the review addresses.	Page 3, line 156~ Page 4, lines 159-162.
**METHODS**			
Eligibility criteria	5	Specify the inclusion and exclusion criteria for the review and how studies were grouped for the syntheses.	Page 4, lines 184-200.
Information sources	6	Specify all databases, registers, websites, organisations, reference lists and other sources searched or consulted to identify studies. Specify the date when each source was last searched or consulted.	Page 4, lines 164 ~ Page 5, lines 210-226.
Search strategy	7	Present the full search strategies for all databases, registers and websites, including any filters and limits used.	Page 4, lines 164-182.
Selection process	8	Specify the methods used to decide whether a study met the inclusion criteria of the review, including how many reviewers screened each record and each report retrieved, whether they worked independently, and if applicable, details of automation tools used in the process.	Page 4, lines 164 ~ Page 5, lines 210-226. Figure 1.
Data collection process	9	Specify the methods used to collect data from reports, including how many reviewers collected data from each report, whether they worked independently, any processes for obtaining or confirming data from study investigators, and if applicable, details of automation tools used in the process.	Page 4, lines 205-214.
Data items	10a	List and define all outcomes for which data were sought. Specify whether all results that were compatible with each outcome domain in each study were sought (e.g. for all measures, time points, analyses), and if not, the methods used to decide which results to collect.	Page 4, lines 206-214.
	10b	List and define all other variables for which data were sought (e.g. participant and intervention characteristics, funding sources). Describe any assumptions made about any missing or unclear information.	Page 4, lines 206-214.
Study risk of bias assessment	11	Specify the methods used to assess risk of bias in the included studies, including details of the tool(s) used, how many reviewers assessed each study and whether they worked independently, and if applicable, details of automation tools used in the process.	Page 5, lines 215-226.
Effect measures	12	Specify for each outcome the effect measure(s) (e.g. risk ratio, mean difference) used in the synthesis or presentation of results.	Page 5, lines 237~ 262.
Synthesis methods	13a	Describe the processes used to decide which studies were eligible for each synthesis (e.g. tabulating the study intervention characteristics and comparing against the planned groups for each synthesis (item #5)).	Page 4, lines 164 ~ Page 5, lines 210-226. Figure 1.
	13b	Describe any methods required to prepare the data for	Not applicable

Section and Topic	Item #	Checklist item	Location where item is reported
		presentation or synthesis, such as handling of missing summary statistics, or data conversions.	
	13c	Describe any methods used to tabulate or visually display results of individual studies and syntheses.	Figures 3 and 4. Supplementary Figures 2 and 3.
	13d	Describe any methods used to synthesize results and provide a rationale for the choice(s). If meta-analysis was performed, describe the model(s), method(s) to identify the presence and extent of statistical heterogeneity, and software package(s) used.	Page 5, lines 227-262.
	13e	Describe any methods used to explore possible causes of heterogeneity among study results (e.g. subgroup analysis, meta-regression).	Page 5, lines 227-262.
	13f	Describe any sensitivity analyses conducted to assess robustness of the synthesized results.	This is not applicable due to the limited number of studies per each outcome.
Reporting bias assessment	14	Describe any methods used to assess risk of bias due to missing results in a synthesis (arising from reporting biases).	Not applicable, did not include any missing results in a synthesis.
Certainty assessment	15	Describe any methods used to assess certainty (or confidence) in the body of evidence for an outcome.	Page 5, lines 227-262.
**RESULTS**			
Study selection	16a	Describe the results of the search and selection process, from the number of records identified in the search to the number of studies included in the review, ideally using a flow diagram.	Page 6, lines 263-273. Figure 1.
	16b	Cite studies that might appear to meet the inclusion criteria, but which were excluded, and explain why they were excluded.	Figure 1.
Study characteristics	17	Cite each included study and present its characteristics.	Pages 6-7. Table 1AB and Supplementary Table 4.
Risk of bias in studies	18	Present assessments of risk of bias for each included study.	Page 12, Tables 3-4, Figure 2, and Supplementary Figure 1.
Results of individual studies	19	For all outcomes, present, for each study: (a) summary statistics for each group (where appropriate) and (b) an effect estimate and its precision (e.g. confidence/credible interval), ideally using structured tables or plots.	Pages 12-18.
Results of syntheses	20a	For each synthesis, briefly summarise the characteristics and risk of bias among contributing studies.	Pages 6-7. Table 1AB and Supplementary Table 4.
	20b	Present results of all statistical syntheses conducted. If meta-analysis was done, present for each the summary estimate and its precision (e.g. confidence/credible interval) and measures of statistical heterogeneity. If comparing groups, describe the direction of the effect.	Pages 12-18.
	20c	Present results of all investigations of possible causes of heterogeneity among study results.	Page 13, Table 3.
	20d	Present results of all sensitivity analyses conducted to assess the robustness of the synthesized results.	This is not applicable due to the limited number of studies per each outcome.

Section and Topic	Item #	Checklist item	Location where item is reported
Reporting biases	21	Present assessments of risk of bias due to missing results (arising from reporting biases) for each synthesis assessed.	Not applicable, did not include any missing results in a synthesis
Certainty of evidence	22	Present assessments of certainty (or confidence) in the body of evidence for each outcome assessed.	Pages 12-18.
**DISCUSSION**			
Discussion	23a	Provide a general interpretation of the results in the context of other evidence.	Pages 19-20.
	23b	Discuss any limitations of the evidence included in the review.	Page 21.
	23c	Discuss any limitations of the review processes used.	Page 21.
	23d	Discuss implications of the results for practice, policy, and future research.	Page 21.
**OTHER INFORMATION**			
Registration and protocol	24a	Provide registration information for the review, including register name and registration number, or state that the review was not registered.	Page 4, lines 166-169.
	24b	Indicate where the review protocol can be accessed, or state that a protocol was not prepared.	Page 22, lines 674-677.
	24c	Describe and explain any amendments to information provided at registration or in the protocol.	Not applicable.
Support	25	Describe sources of financial or non-financial support for the review, and the role of the funders or sponsors in the review.	Page 22, lines 655-670.
Competing interests	26	Declare any competing interests of review authors.	Page 22, line 679.
Availability of data, code and other materials	27	Report which of the following are publicly available and where they can be found: template data collection forms; data extracted from included studies; data used for all analyses; analytic code; any other materials used in the review.	Page 22, lines 674-677.

From: Page MJ, McKenzie JE, Bossuyt PM, Boutron I, Hoffmann TC, Mulrow CD, et al. The PRISMA 2020 statement: an updated guideline for reporting systematic reviews. BMJ 2021;372:n71. doi: 10.1136/bmj.n71

Supplementary Table S2. Search Strategies

PubMed: ("colorectal cancer"[Mesh] OR "colon cancer" OR "rectal cancer" OR "CRC" OR "colorectal neoplasms" OR "colorectal adenocarcinoma") AND ("chemotoxicity"[Mesh] OR "chemotherapy" OR "chemotherapeutic agents" OR "antineoplastic agents") AND ("risk factors"[Mesh] OR "predictive models" OR "prognostic factors" OR "response prediction") AND (English[lang] AND "adult"[MeSH])

Cochrane Library:

#1 MeSH descriptor: [Colorectal Neoplasms] explode all trees
#2 MeSH descriptor: [Chemotoxicity] explode all trees
#3 MeSH descriptor: [Risk Factors] explode all trees
#4 (#1 AND #2 AND #3)

Web of Science:

TI=("colorectal cancer" OR "CRC" OR "colorectal neoplasms" OR "colorectal adenocarcinoma") AND TI=("chemotoxicity" OR "chemotherapy" OR "chemotherapeutic agents" OR "antineoplastic agents") AND TI=("risk factors" OR "predictive models" OR "prognostic factors" OR "response prediction")

CINAHL:

(AB ((MH "Colorectal Neoplasms+") OR "colorectal cancer" OR "CRC" OR "colorectal neoplasms" OR "colorectal adenocarcinoma") AND AB ((MH "Chemotoxicity+") OR "chemotoxicity" OR "chemotherapy" OR "chemotherapeutic agents" OR "antineoplastic agents") AND AB ((MH "Risk Factors+") OR "risk factors" OR "predictive models" OR "prognostic factors" OR "response prediction"))

EMBASE:

('colorectal neoplasm'/exp/mj OR 'colorectal cancer'/mj OR 'CRC'/mj OR 'colorectal neoplasms'/mj OR 'colorectal adenocarcinoma'/mj) AND ('chemotoxicity'/exp/mj OR 'chemotherapy'/mj OR 'chemotherapeutic agents'/mj OR 'antineoplastic agents'/mj) AND ('risk factors'/exp/mj OR 'predictive models'/mj OR 'prognostic factors'/mj OR 'response prediction'/mj) AND ([article]/lim OR [article in press]/lim) AND [english]/lim AND ([embase]/lim OR [embase classic]/lim)

PsycINFO:

ab("colorectal cancer" OR "CRC" OR "colorectal neoplasms" OR "colorectal adenocarcinoma") AND ab("chemotoxicity" OR "chemotherapy" OR "chemotherapeutic agents" OR "antineoplastic agents") AND ab("risk factors" OR "predictive models" OR "prognostic factors" OR "response prediction")

Supplementary Table S3. Quality Assessment by Critical Appraisal Skills Program (CASP) Checklist for Studies Included for Systematic Review and Meta-Analysis (N =30)

Author(s), Year.	Q 1	Q 2	Q 3	Q 4	Q 5	Q 6	Q 7	Q 8	Q 9	Q 10	Q 11	Q 12	Final Quality (L/M/H)
**Observational Cohort Studies reviewed by the CASP Cohort Study Checklist 12 Qs**													
Ali et al. (2016)	Y	Y	Y	Y	Y	Y	Y	Y	Y	Y	Y	Y	H
Antonio et al. (2018)	Y	Y	Y	Y	Y	N	Y	Y	Y	N	Y	Y	H
Aparicio et al. (2016)	Y	Y	Y	Y	Y	Y	Y	Y	Y	Y	Y	Y	H
Backshall et al. (2011)	Y	Y	Y	Y	Y	Y	Y	Y	Y	Y	Y	Y	H
Barret et al. (2011)	Y	Y	Y	Y	Y	Y	Y	Y	Y	Y	Y	Y	H
Beukers et al. (2021)	Y	Y	Y	Y	Y	N	Y	Y	Y	Y	Y	Y	H
Breton et al. (2021)	Y	Y	Y	Y	Y	Y	Y	Y	Y	Y	Y	Y	H
Brown et al. (2022)*	Y	Y	Y	Y	Y	Y	Y	Y	Y	Y	Y	Y	H
Cespedes Feliciano et al. (2017)*	Y	Y	Y	Y	Y	Y	Y	Y	Y	Y	Y	Y	H
Decoster et al. (2018)*	Y	Y	N	Y	Y	Y	Y	Y	Y	Y	N	Y	H
Feliu et al. (2022)	Y	Y	Y	Y	Y	Y	Y	Y	Y	Y	N	Y	H
Folprecht et al. (2008)	Y	Y	Y	Y	Y	Y	Y	Y	Y	Y	Y	Y	H
Gallois et al. (2019)	Y	Y	Y	Y	Y	Y	Y	Y	Y	Y	Y	Y	H
Garg et al. (2012)	Y	Y	Y	Y	Y	Y	Y	Y	Y	Y	Y	Y	H
Grimes, C. (2022)*	Y	Y	Y	Y	Y	N	Y	Y	Y	Y	Y	Y	H
Hochster et al. (2007)	Y	Y	Y	Y	Y	Y	Y	Y	Y	Y	Y	Y	H
Jung et al. (2015)	Y	Y	Y	Y	Y	Y	Y	Y	Y	Y	Y	Y	H
Karabulut et al. (2022)	Y	Y	Y	Y	Y	N	Y	Y	Y	Y	Y	Y	H
Li et al. (2021)	Y	Y	Y	Y	Y	Y	Y	Y	Y	Y	Y	Y	H
Looijaard et al. (2020)*	Y	Y	Y	?	Y	N	Y	Y	Y	Y	Y	Y	H
Okada et al. (2017)	Y	Y	Y	Y	Y	Y	Y	Y	Y	Y	Y	Y	H
Retornaz et al. (2020)	Y	Y	N	Y	Y	?	Y	Y	Y	Y	Y	Y	H
Sastre et al. (2012)	Y	Y	N	Y	?	Y	Y	Y	Y	Y	Y	Y	H
Seymour et al. (2011)	Y	Y	Y	Y	Y	N	Y	Y	Y	Y	Y	Y	H
Stein et al. (2016)	Y	Y	Y	Y	Y	N	Y	Y	Y	Y	Y	Y	H
Tominga et al. (2016)	Y	Y	N	Y	Y	Y	Y	Y	Y	Y	Y	Y	H
Tsuchihashi et al. (2018)	Y	Y	Y	Y	Y	N	Y	Y	Y	Y	Y	Y	H
Watanabe et al. (2018)	Y	Y	N	Y	N	?	Y	Y	Y	Y	Y	Y	H
Yamada et al. (2013)	Y	Y	N	Y	Y	Y	Y	Y	N	Y	Y	Y	H
**Randomized Controlled Trial (RCT) reviewed by the CASP RCT Checklist 11 Qs**													
Osterlund et al. (2007)	Y	Y	Y	Y	Y	Y	Y	Y	Y	Y	Y	Not applicable	H

Note. Responses: Yes (Y), Can't tell (?), No (N). Article Quality Rating: High (H); Medium (M); Low (L). Using an arbitrary threshold, we evaluated an article as "high" quality if it met at least 80% of the checklist criteria (e.g., 10 of 12 questions in each study), "low" quality if it met 50% or less of the criteria, and "medium" quality if it met > 50% and < 80% of the criteria.

CASP checklist for cohort studies: Question (Q) 1 (Clear Aims/Research Questions), Q 2 (Study design & recruitment), Q 3 (Exposure accurately measured), Q 4 (Outcomes accurately measured), Q 5 (Identified confounders), Q 6 (Appropriate subject follow-up), Q 7 (Clear results), Q 8 (How precise are the results), Q 9 (Reliability of the results), Q 10 (Generalizability), Q 11 (Results fit with other available evidence), Q 12 (Clinical Implications)

CASP checklist for RCT studies: Q 1 (Clear Aims/Research Questions), Q 2 (Study design & recruitment, randomization), Q 3 (All participants accounted for at its conclusion), Q 4 (Blinding), Q 5 (Group homogeneity), Q 6 (Treatment equity), Q 7 (Clear results), Q 8 (Precision of the estimate of the intervention effect), Q 9 (Benefits versus Harms), Q 10 (Generalizability to local population), Q 11 (Additional value of the current intervention compared to the existing interventions).

*5 studies were excluded from meta-analyses due to unavailable chemotoxicity prevalence data or data that was not comparable for inclusion.

Supplementary Table S4. Detailed characteristics of Studies included for Systematic Review and Meta-Analysis (N =30)

Authors/year/ Country	Samples N/Sex/Age/Race/Stages/ Primary Chemotherapy/ Study Design	Time points of measuring toxicity /Measures/Prevalences of chemotoxicity	Significant Risk factors of chemotoxicity	Others
Ali et al. (2016)/ Canada and France	N= 138 patients with CRC (colon only 70%)<br><br>F (50%)<br>Race no data reported (80 Canadian and 58 French)<br><br>Stages: Mixed, I (1.5%), II (6%), III (20%), IV (72.5%)<br><br>Mean age: 61.5 (±10.3: 28-87)<br><br>5-FU-based agents (95%) /Prospective longitudinal study	After each cycle up to 12 cycles/ CTCAE/<br>After 12 cycles of chemotherapy data<br>-Overall moderate-to-severe toxicity (45.3%).<br>-Non-hematological toxicity (38.7%): GI toxicity (20%), neuropathy (13%)<br>-Hematological toxicity (3.6%): anemia (1.4%), neutropenia (2.2%).<br><br>(No available data of mild toxicity prevalence)	Low body mass is a significant predictor of toxicity and neuropathy in patients administered FOLFOX-based regimens.<br><br>(no AOR was computed).	31.2% of patients who experienced chemotoxicity delayed chemotherapy or reduced doses of chemotherapy.
Antonio et al. (2018)/ Spain	N= 193 CRC (colon only 74%)<br><br>F (32%),<br>Race (no data reported)<br>Mean age: 80 (±5: 75-89)<br>Stages: II (28%) III (72%).<br><br>5-FU single or combined with other agents (50%), capecitabine single or combined with other agents (50%)/ Prospective study	After completion of chemotherapy/CTCAE<br><br>-Non-hematological toxicity moderate-to-severe (24.3%): fatigue (8.1%), anorexia (2.7%), diarrhea (9.5%), stomatitis (2.7%), neuropathy (8.1%).<br>-Hematological toxicity moderate-to-severe (5.4%): neutropenia (5.4%)<br><br>(No available data of mild toxicity prevalence)	Age, sex, cancer site, tumor stage, social support, geriatric status, and weight loss were not associated with toxicity.<br>Comprehensive geriatric assessment-based classification was significantly associated with chemotoxicity prevalence.	Among the multi-variate analysis, the chemotoxicity was the only significant predictor of incompletion of the chemotoxicity: 7.2 times higher risk of chemotherapy incompletion in patients reporting moderate-to-severe toxicity.
Aparicio et al. (2016) /France	N= 271 mCRC (no data on colon or rectum cancers)<br><br>F (42.4%)<br>Race (no data reported)<br>Stage: IV	After receiving at least one dose of chemotherapy.<br>The median time of onset of moderate-to-severe toxicity was 6-7months of 5-FU primary-based chemotherapy versus 2 months mixed with irinotecan/CTCAE/<br><br>Moderate-to-severe toxicity (65%).	No data of ORs or risk factors.	Even though mild chemotoxicity, the prevalence of mild chemotoxicity is high and needs to be managed.

	Mean age: 80 (±5; range 85-92), All ≥ 75 y.o.)<br><br>5-FU based agents (single or combined with other regimens)/ Prospective study	-Non-hematological (53%):<br>nausea (4%), vomiting (4%), diarrhea (14%), thromboembolic events (6%), mucositis (1.5%), cardiotoxicity (1.5%).<br>-Hematological (24%):<br>anemia (3%), neutropenia (22%).<br><br>Mild toxicity (34.3%).<br>-Non-hematological (43%):<br>nausea (46%), vomiting (31%), diarrhea (36%), thromboembolic event (2%), mucositis (23.5%), cardiac toxicity (0%)<br>-Hematological (35%):<br>anemia (82%), neutropenia (33%).		
Backshall et al. (2011)/ United Kingdom	N=52 mCRC<br>Colon only (73%), rectal (14%).<br><br>F (35%)<br>Race (no data reported)<br>Stages: locally advanced or metastatic colorectal cancer.<br><br>Capecitabine (100%)/ Prospective study	During the 1st cycle of chemotherapy/ CTCAE /<br><br>Moderate-to-severe toxicity (11%):<br>-Non-hematological toxicity:<br>fatigue (2%), hand-foot syndrome (11%), diarrhea (9%), nausea/vomiting (2%).<br>-Hematological toxicity (10%)<br><br>Mild toxicity<br>-Non-hematological toxicity (25%):<br>fatigue and diarrhea (25%), hand-foot syndrome (11%), nausea/vomiting (9%).<br>-Hematological toxicity (17%): anemia 13%, abnormal lipid panel 4%	Serum metabolites of lipid profiles.<br>Higher levels of low-density lipoprotein–derived lipids, including polyunsaturated fatty acids and choline phospholipids, predicted higher-grade toxicity over the treatment period.	Diarrhea was the most significant factor in treatment discontinuation.<br><br>Even though the patients reported low-level severity, 25% reported fatigue and diarrhea, which should not be ignored for management.
Barret et al. (2011)/ France	N=114 mCRC (Colon only 70%)<br><br>F (31.6%),<br>Race (no data reported)<br>Stages: Metastatic (100%).<br><br>Mean age: 65 (±15: 22-92)<br><br>5-FU single or combined with other agents (100%) / Prospective study	During the 2 months following chemotherapy /CTCAE<br><br>-Non-hematological toxicities moderate-to-severe (22.3%): GI toxicity (28.7%), neuropathy (85.5%) (8.1%).<br>-Hematological toxicity moderate-to-severe (38.3%).	Malnutritional status measured by albumin, weight loss<br>Predicted overall GI and hematological toxicity, but not peripheral neuropathy AOR: 13.5 (1.1–169.3)<br>Albumin biomarker was included.	Risk factors of chemotoxicity might be different per types of hematological and non-hematological toxicity.

		(No available data of mild toxicity prevalence)		
Beukers et al. (2021) / Netherlands	N = 97 colon only cancer (100%)<br><br>F (52%)<br>Race (no data reported)<br>Stages: II (22.7%), III (77.3%).<br><br>Mean age: 77.2 (±4.8: range 70-85, all ≥ 70 y.o.).<br><br>Capecitabine (100%) /Retrospective study	25% frail/Moderate-to-severetoxicity (CTCAE) 3-6 months after chemotherapy.<br><br>Moderate-to-severe toxicity (48%)<br>-Non-hematological<br>GI, including nausea, vomiting, diarrhea (30%), hand-foot syndrome (20%), other including fatigue, rash, and mucositis (1.4%).<br>-Hematological (5%).<br><br>(No available data of mild toxicity prevalence)	In multivariable analyses, female sex (AOR 2.33, 0.87-6.24) tumor stage II (AOR 4.84, 1.31-17.51), postoperative complications (AOR 3.8, 1.37-10.53), and frailty status (AOR 4.21, 1.04-17.17).<br>no specific biomarkers	No sensitivity and specificity of the prediction regression model.
Breton et al. (2021) / France	N = 2,190 MCRC (colon only 75%).<br><br>F (38.4%)<br>Race (no data reported)<br>Stage II (22.7%), III (77.3%).<br><br>Mean age: 66.8 (±8: range 58.9-75.01)<br><br>5-FU single or combined with other agents (100%)/<br>Prospective study	Toxicity was defined as the occurrence of grade III toxicity within 3 months after initiation of Chemotherapy/ CTCAE<br><br>Moderate-to-severe toxicity (52.2%).<br>-Non-hematological toxicity (45.3%):<br>GI toxicity (15.0%), including diarrhea (6%), nausea-vomiting (4%), mucositis (3.4%), indigestion/abdominal pain (3.6%), and appetite loss (1.1%); fever (0.5%); thrombosis (2.6%); infection (3.0%); respiratory-cardio-renal disorders, fatigue and psychological problems, HEENT disorders, musculoskeletal disorders (all less than <1.0%).<br>-Hematological toxicity (19.9%): neutropenia (13.2%), leukocytosis (high WBC, 4.6%), liver enzyme elevation (0.4%)<br><br>(No available data of mild toxicity prevalence)	Prognostic factors for an early grade III toxicity (ET3) in multivariate analysis were an ECOG performance status, Tri therapy versus monotherapy, alkaline phosphatase > 300 UI/l, and no primary tumor resection.<br><br>Age, sex, performance status, BMI, CEA, CA19-9, liver panels, Creatinine, Hb, WBC, Platelet.	Overall survival in patients without early toxicity was significantly longer than that in patients with toxicity (13% higher rates of mortality).<br>No measure of frailty,<br>The onset of early toxicity may require dose adjustments (Relative Dose Intensity). However, this can inhibit the efficacy of cancer treatments. Thus, determining the predictors of severe early toxicity can influence the treatment choice and allow the optimization of supportive care from the beginning of the treatment.

Feliu et al. (2022) / Spain	N= 215 CRC<br>(no data on colon or rectal cancers)<br><br>F (32%)<br>Race (no data reported)<br>Stages: I-III (51%), IV (49%)<br><br>Mean age: 78 (±4.9; range 70-90, all ≥ 70 y.o.)<br><br>5-FU based agents (37%), capecitabine (77%)/<br>Prospective study, one-time measure	At 6 months post-chemotherapy initiation/CTCAE/<br><br>-Overall moderate-to-severe toxicity (54%).<br>-Non-hematologic toxicity moderate-to-severe (28%): fatigue (12%), diarrhea (10%).<br>-Hematologic toxicity moderate-to-severe (17%): neutropenia (8%) and anemia (6%).<br><br>-22% death due to chemotoxicity combined with cancer progression.<br>-16% unplanned hospitalization due to toxicity primarily, febrile neutropenia and sepsis, and diarrhea.<br><br>Mild overall chemotoxicity (66%)	Risk factors:<br>Frailty (75%), Factors related to the development of moderate-to-severe toxicity were IADL ≤ 7, creatinine clearance ≤ 50 mL/min, unintentional weight loss during the treatments ≥ 5%<br><br>Weight loss B: 0.71 (SE 0.3), HR 2.01 (1.14-4.21)<br>IADL B: 0.46 (0.3), HR 1.29 (1.01-2.31)<br>Creatinine clearance rate <50/min B: 0.63 (0.3)<br>HR 1.89 (1.06-3.38)	A combination of geriatric and clinical characteristics can better predict the risk of severe chemotoxicity. These measures should start before the initiation of chemotherapy and should be kept over time, with frequent patient reevaluation, instead of treating after experiencing chemotoxicity.
Folprecht et al. (2008) /UK.	N= 2,691 CRC<br>(no data on colon or rectum cancers)<br><br>F (33%)<br>Race (no data reported)<br>Stages: IV (50%)<br><br>Mean age: 70 (±5; range 18-79)<br><br>5-FU-based agents and 50% oxaliplatin/<br>Prospective study	During the first month of the treatment/CTCAE/<br><br>Moderate-to-severe overall toxicity (average 28%).<br>-Non-hematological toxicity moderate-to-severe: diarrhea (20.5%), vomiting (9.6%), nausea (11.3%), neuropathy (1%), thrombosis (4.9%), hepatic toxicity (4.6%).<br>-Hematological toxicity moderate-to-severe: leukopenia (16.9%), neutropenia (28.9%).<br><br>(No available data of mild toxicity prevalence)	Age differences were unrelated to toxicity (cut off = 70 years old).<br>white blood cell count (≤ 10 *v* > 10 × 10^9/L), level of alkaline phosphatase (≤ 300 *v* > 300 U/L), and lactate dehydrogenase level (≤ 250 *v* > 250 U/L) were associated with overall toxicity.	No risk factors were tested. Chronological age might not be a sensitive predictor of chemotoxicity.
Gallois et al. (2019) /France	N=168 mCRC<br>(no data on colon or rectum cancers)<br><br>F (44%), Race (no data reported)<br>Stages: Metastatic (100%).	During the chemotherapy (from start to 2 months)/CTCAE/<br><br>Moderate-to-severe overall toxicity (26%): nausea/vomiting (15%), diarrhea (8%), mucositis (4%), hand-	In multivariate analysis, malnutrition and functional status were significantly associated with chemotherapy-related grade => clinical toxicities	Age itself was not a significant factor in chemotoxicity. Either functional status or nutritional status were

	Mean age: 75 (±5: 70-92)<br>5-FU single or combined with other agents (96%)/ Prospective study	foot skin reactions (0.6%, and alopecia (4%).	(malnutrition AOR 3.7; 1.7-8.4).<br>Included biomarkers such as lymphocytes, Hb, albumin, but no variable of geriatric assessment.	significant factors of chemotoxicity.
Garg et al. (2012)/ Australia	N= 173 patients with CRC (no data on colon or rectum cancers)<br><br>F (43%)<br>Race (no data reported)<br>Stages: II (15%), III (85%)<br><br>Mean age: 63 (± 5:54-72)<br><br>5-FU-based agents (100%) /Prospective longitudinal study	At the end of the first month of the chemotherapy (Weekly follow-up)/CTCAE/<br><br>-Overall moderate-to-severe toxicity (46%).<br>-Non-hematological toxicity (33%): GI toxicity (50.6%), diarrhea (11%), mucositis/stomatitis (12%),<br>-Hematological toxicity (62%): neutropenia (55%), leukopenia (12%), thrombopenia (2%)<br><br>Mild toxicity<br>-Non-hematological toxicity: diarrhea (83%), mucositis (58%).<br>-Hematological toxicity: neutropenia (43%), leukopenia (40%)	Blood markers, complete blood counts, and telomere length from blood were used.<br>Used Beta coefficient data. Multivariate analysis showed that hematological toxicity was predicted by short telomere length, high platelet lymphocyte ratio (PLR), and low neutrophil count (R^2=0.38, P<0.0006) at baseline. In contrast, mucositis was predicted by younger age, short telomere length, and high PLR (R^2=0.34, P<0.001).	Age itself was not associated with telomere length. Younger age was associated with more toxicity and the need to consider telomere length and biological aging compared to chronological age.
Hochster et al. (2007) /USA	N= 55 CRC (no data on colon or rectum cancers)<br><br>F (47.3%)<br>Race (White 93%, Black 7%)<br>Stages III and IV<br>Mean age: 81 (±4; range 75-90, all $\geq$75 y.o.).<br><br>Leucovorin chemotherapy/ Prospective study	Anytime from baseline to completion of chemotherapy/CTCAE/<br><br>Moderate-to-severe overall toxicity (average 55%)<br>-Non-hematological toxicity diarrhea (25%), fatigue (14.3%), nausea (10.7%), and all other GI toxicities (35.7%).<br>-Hematological toxicity neutropenia (5.4%).<br><br>(No available data of mild toxicity prevalence)	Age was associated with specifically GI toxicity (age older than 85 years old was at highest risk).<br>ECOG performance status, Race was not a significant factor for chemotoxicity.<br>No relationship tests were examined.<br>Biomarkers: CEA, liver panel, Creatine.	Further suggestions of age-related factors, including age-related biomarkers and functional status, are suggested.
Jung et al.(2015) / South Korea	N= 229 patients<br>Colon only cancer (100%)	During the 12 cycles of chemotherapy (biweekly)/CTCAE/	Recorded moderate-to-severe adverse events (45%	BMI was a confounder (associated with muscle mass), but this can be

	F (58.5%)<br>Race (no data reported)<br>Stages: I (0.9%), II (2.2%), III (74.6%), IV (22.3%).<br><br>Mean age 61 (±3: 53-67)<br><br>5-FU based agents (100%)/ Prospective study	Baseline muscle mass was assessed by measuring the cross-sectional area of the psoas muscle at the level of the fourth lumbar vertebra on computed tomography image (psoas muscle mass index)<br><br>(No available data of mild toxicity prevalence)	experienced) included neuropathy, neutropenia, anemia, thrombocytopenia, nausea, vomiting, diarrhea, mucositis, and liver function abnormalities. moderate-to-severe neutropenia (40% reported) was the most prevalent toxicity. (no data available for individual toxicity prevalence)<br><br>Psoas muscle mass measured by CT was used to predict chemotoxicity. Moderate-to-severe toxicities (AOR=1.56, 95 % CI=1.05–2.38 per 1 standard deviation decrease in the psoas muscle)	controversial as BMI was positively associated with higher chemotherapy dose absorption rate with increased toxicity.<br>Thus, multiple nutritional factors need to be considered in chemotherapy prediction models.<br>models.
Karabulut et al. (2022)/Turkey	N=137 mCRC<br>(no data on colon or rectum cancers)<br><br>F (61%)<br>Race (no data reported)<br>Stages: Metastatic cancer (100%).<br><br>Mean age:62 (±8: 18-83)<br><br>5-FU single or combined with other agents (100%)/ Prospective study	After the first line of the chemotherapy cycle/CTCAE/<br><br>-Non-hematological toxicity moderate-to-severe (43%): GI toxicity (15.5%), diarrhea (10%), nausea/vomiting (14%), constipation (3%), neuropathy (14%).<br>-Hematological toxicity moderate-to-severe (45%): leucopenia (35%), anemia (30%).	Nutrition<br>Albumin, BMI, weight loss<br><br>Biomarkers (CEA, CA19-9, albumin)<br><br>Moderate/severe malnutrition was associated with more cytopenia, nausea/vomiting, diarrhea, and neuropathy ($p < 0.05$ for all parameters). No data of OR.	In mCRC patients, moderate/severe malnutrition is associated with worse non-hematological toxicities.
Li et al. (2021)/China	N= 233 patients with CRC<br>(no data on colon or rectum cancers)<br><br>F (34%)<br>Race: no data reported (233 Chinese)	From first to 8 cycles, follow up at each cycle/CTCAE/<br>After 8 cycles of chemotherapy data<br>-Overall moderate-to-severe toxicity (35%).<br>-Non-hematological toxicity (40%): nausea/vomiting (35%), hand-foot	Age (AOR 1.15; 1.03-1.35, p=.035), WBC increased change (AOR 0.53; 0.26-0.87, p =.036) and nausea.<br>Age was associated with neutropenia (AOR 0.96; 0.93-0.99, p = .025), and	Baseline low values of WBC, neutrophil, RBC, hemoglobin, and PLT were associated with higher toxicity (more specific to hematological toxicity).

	Stages: Mixed (no accurate data)<br><br>Mean age: 58 (±10.5: 28-87)<br><br>Capecitabine (100%)/ Prospective longitudinal study	syndrome (20%), abdominal pain (5%), diarrhea (5%), constipation (3%)<br>-Hematological toxicity (25%), including anemia, neutropenia, and thrombocytopenia.<br><br>(No available data of mild toxicity prevalence)	anemia (AOR 1.04; 1.01-1.09, p =.023).<br><br>Weight (AOR 0.89; 0.80-0.97, p =.017 with hand-foot syndrome), (AOR 0.92; 0.84-0.98, p =.034) with nausea.<br>Baseline hemoglobin albumin, and CRP were associated with anemia toxicity.<br>Weight, BMI, Albumin, platelet, RBC, Hb, WBC, and CRP were included.	High CRP, PLR, and lymphocyte at baseline were associated with GI toxicity and non-hematological toxicity. Chemotherapy leads to altered inflammatory response, and inactive immune function regeneration function contributes to the susceptibility to chemotoxicity.
Okada et al. (2017)/Japan	N=108 mCRC<br>(75% colon only)<br><br>F (56%)<br>Race (no data reported)<br>Stages: (no data reported)<br><br>Mean age: 65 (±9: 34-83).<br>5-FU single or combined with other agents (100%)/<br>Retrospective study	After 6 months of chemotherapy cycle/CTCAE/<br><br>-Non-hematological toxicity moderate-to-severe (57%): no individual symptoms were reported.<br>-Hematological toxicity moderate-to-severe (45%): no individual symptoms were reported.	Albumin, WBC, neutrophil, CRP, CEA, CA19-9, BUN/Cr.<br>No Data of OR<br><br>Based on the nutritional status measured albumin levels at 6 months after chemotherapy were associated with overall toxicity.	Baseline nutritional status was associated with future chemotherapy toxicity, which also was associated with poor overall survival and PFS.
Osterlund et al. (2007)/ Finland	N=150 mCRC<br>Colon only (60%)<br><br>F (49%)<br>Race (no data reported)<br>Stage: Mixed II-IV<br><br>Mean age 60 (31-75).<br><br>5-FU based agents (100%)/ RCT<br><br>(Lactobacillus injection n = 52 group versus control group n = 98)	During chemotherapy from 2 to 6 months/CTCAE/<br>After 6 months of chemotherapy data:<br>-Moderate-to-severe overall toxicity (58%).<br>-Non-hematological toxicity (40%): stomatitis (41%), diarrhea (37%).<br>GI toxicity (51%).<br>-Hematological toxicity (18%): neutropenia (16%), hand-foot syndrome (2%)<br>Mild overall toxicity (65%).-Non-hematological toxicity (80%): diarrhea (75%), hand-foot syndrome (90%).<br>-Hematological toxicity (70%).	Gut microbiome status (but no direct measures modulated by the probiotics intervention.	Patients who received *Lactobacillus* had less grade 3 or 4 diarrhea (22 *vs* 37%, *P*=0.027), reported less abdominal discomfort, needed less hospital care, and had fewer chemotherapy dose reductions due to bowel toxicity.<br>However, lactobacillus was not associated with overall toxicity or hematological toxicity.

Retornaz et al. (2020) / France	N=97 colon cancer<br><br>F (51%)<br>Race (White European)<br>Stage: II-III (46.2%), IV (53.8%)<br><br>Mean age: 79 (±4.5; range 70-90) (all ≥ 70 y.o.)<br><br>5-FU based agents (70%)/ Longitudinal Prospective study, repeated measures	At 3, 6, 9, 12 months post-chemotherapy/CTCAE/<br><br>Moderate-to-severe toxicity (50% experienced during the first 500 days).<br>-Non-hematologic toxicity (44%): fatigue (64%), neuropathy (44%) and GI symptoms (40% average: nausea 43%, diarrhea 34.0%).<br>-Hematologic toxicity (34%): neutropenia (27%), high WBC (21%), low PLT (20%), and anemia (36%)<br><br>Mild chemotoxicity (66% average over the 500 days follow-up post chemotherapy initiation).	Frailty (65%), Metastatic status, polychemotherapy, impaired grip strength, increased C-reactive protein and alkaline phosphatase levels, decreased lymphocyte count, and hypoalbuminemia (nutritional status).<br>Albumin AOR 3.94, polychemotherapy AOR 2.06<br>Grip status AOR 2.18<br>CRP AOR 1.90<br>ECOG-PS AOR 0.43	Multivariate chemo prediction model including geriatric assessment, frailty markers, laboratory data, and oncologic parameters with sensitivity 81.6%, and specificity 71.4%.<br>Limitations include a small sample size and mixed cancer stages.<br><br>-30% death, with 21% of death due to chemotoxicity.
Sastre et al. (2012) /Spain	N= 66 mCRC<br>(no data on colon or rectum cancers)<br><br>F (42.4%), Race (no data reported), Stage: IV<br><br>Mean age: 70 (±7; range 70-86), All ≥ 70 y.o.)<br><br>Cetuximab plus capecitabine / Prospective study	After 6 weeks of treatments/CTCAE/<br><br>Moderate-to-severe toxicity:<br>-Non-hematological toxicity: paronychia (15%), rash (28%), neuropathy (21%), diarrhea (16%), respiratory (7.5%), GI toxicity (16%)<br>-Hematological toxicity: thrombosis (3.7%).<br>(No available data of mild toxicity prevalence)	No data on ORs or risk factors.	The majority of chemotoxicity included non-hematological toxicity.
Seymour et al. (2011) /UK	N= 440 mCRC<br>colon (78%), rectum (22%)<br><br>F (41%)<br>Race (no data reported)<br>Stage: IV<br><br>Mean age: 74 (±5; range 35-86).<br><br>5-FU-based agents (50%) versus Capecitabine-based agents (50%)/ Prospective study	After 1-12 weeks of chemotherapy/CTCAE/<br><br>Non-hematological toxicity moderate- to severe (52%):<br>nausea (4%), anorexia (4%), stomatitis (2%), diarrhea (10%), fatigue (9%), pain (16%), neuropathy (1%)<br>-Hematological toxicity moderate-to-severe (6%): anemia (3%), neutropenia (3%)<br><br>Mild toxicity (36%)	Performance status, WBC, quality of life score at baseline, and overall toxicity were associated with end-point treatment efficacy.	The outcome was treatment efficacy (i.e., response).<br>Efficacy measures are weighed against toxic effects, convenience, and other variables before deciding which treatment is best.

		-Non-hematological toxicity (75%): anorexia (15%), stomatitis (10%), diarrhea (24%), fatigue (39%), pain (19%), neuropathy (8%)<br>-Hematological toxicity (24%): anemia 17%), neutropenia (7%)		
Stein et al. (2016)/Germany	N= 1,249 patients with mCRC (no data on colon or rectum cancers)<br><br>F (45%)<br>Race (no data reported)<br>Stages: Metastasis<br><br>Mean age: 74 (± 12:21-99)<br><br>Capecitabine (100%)/ Prospective longitudinal study	After each cycle up to 8 cycles/ CTCAE/ After 8 cycles of chemotherapy data data<br>-Overall moderate-to-severe toxicity (37%).<br>-Non-hematological toxicity (55%): nausea (8%), vomiting (6%), diarrhea (12%), pain (12%), fever (0%), mucositis/stomatitis (4%), neuropathy (12%).<br>-Hematological toxicity (23%): anemia (12%), neutropenia (6%), leukopenia (5%), thrombopenia (7%).<br><br>(No available data of mild toxicity prevalence)	75 years old age cut-off was used as a risk factor.<br>Severe toxicities occurred rarely without any difference regarding age groups.	This study did not assess functional status; therefore, functional status may be more important than age itself.<br><br>To stratify elderly patients to the different treatment intensities, a comprehensive geriatric assessment should be applied.
Tominga et al. (2016)/Japan	N=136 CRC (no data on colon or rectum cancers)<br><br>F (58%)<br>Race (no data reported)<br>Mean age: 63 (±6: 58-71)<br>Stages: III (100%).<br><br>Capecitabine, and 5-FU (95%)/ Retrospective study	After the completion of later-line chemotherapy cycles /CTCAE/<br><br>-Non-hematological toxicity moderate-to-severe: Anorexia (17%), diarrhea (7.5%).<br>-Hematological toxicity moderate-to-severe (37%): Neutropenia (52%).<br><br>Mild overall chemotoxicity (63%): no individual symptoms were reported.	CRP/albumin ratio was associated with severe chemotoxicity. High levels of the Glasgow Prognostic Score (GPS) and the neutrophil-to-lymphocyte ratio (NLR) appeared to be associated with the CRP and albumin ratio. CAR≥0.1 (HR: 7.06, 95% CI: 2.51–19.88, p<0.01) as a significant determinant of severe side effects of AC.	The present study showed that the CAR is a novel and promising inflammation-based score for ≥ grade 3 side effect
Tsuchihashi et al. (2018) /Japan	N=523 mCRC (no data on colon or rectum cancers)<br><br>F (41%); Race (no data reported)<br>Stage: metastatic (100%)	After the completion of later-line chemotherapy cycles /CTCAE/<br><br>Non-hematological toxicity moderate-to-severe(30%):	CEA, BMI, and The Glasgow Prognostic Score (GPS) were proposed as an objective indicator reflecting a patient’s systemic nutritional and inflammatory	Unique chemotoxicity is more likely for liver dysfunction and skin issues, not primarily with GI toxicity.

	Mean age: 63 (±9: 55-85)<br><br>Regorafenib, trifluridine/tipiracil/ Retrospective study	fatigue (3%), anorexia (6%), hand footskin reaction (20%), liver dysfunction (10%), skin disorder (4%).<br>-Hematological toxicity moderate-to-severe (22%):<br>neutropenia (11%), anemia (12%).	status, but these risk factors were not tested with chemotoxicity.	
Watanabe et al. (2018)/Canada	N= 371 patients with CRC (no data of colon or rectum cancers).<br><br>F (49%)<br>Race (no data reported)<br>Stages: III (100%)<br><br>Mean age: 64 (±5: 60-89)<br><br>5-FU-based agents (59%) or capecitabine (41%)/ Prospective longitudinal (two-time points)	Baseline and at 12 weeks after chemotherapy. Monthly follow up/CTCAE<br>After chemotherapy data<br>-Overall moderate-to-severe toxicity (78%.)<br>-Non-hematological toxicity (45%): GI toxicity (80%), neuropathy (80%).<br>-Hematological toxicity (40%): anemia, neutropenia, and thrombocytopenia.<br><br>(No available data of mild toxicity prevalence)	Age (≥ 70) (OR 3.30; 95% CI 1.17–9.37; P = 0.025), pre-treatment anemia (OR 23.18; 95% CI 6.36–84.48; P < 0.001), female sex (OR 5.13; 95% CI 2.08–12.68; P < 0.001). GI toxicities were less likely to occur among tumors at the distal area versus the proximal area (OR 0.38; 95% CI 0.15–0.99; P = 0.047). Also included comorbidities and ECOG PS.	Authors suggested the use of relative factors of chemotoxicity in cancer treatment decision-making, specifically for older adults.
Yamada et al. (2013)/Japan	N= 512 mCRC (no data on colon or rectum cancers)<br><br>F (36%)<br>Race (no data reported)<br>Stages: IV (100%)<br>Mean age: 63 (±5; range 33-79).<br><br>5-FU-based agents (50%) and oxaliplatin (50%)/<br>Prospective study	After the first line of chemotherapy /CTCAE/<br><br>-Non-hematological toxicity moderate-to-severe: anorexia (6%), diarrhea (12%), GI sepsis including obstruction or constriction, and GI perforation (9%).<br>-Hematological toxicity moderate-to-severe: leucopenia (10%), neutropenia (43%)<br><br>(No available data of mild toxicity prevalence)	No data on ORs or risk factors.	Critical findings of high prevalence and severity of GI toxicity.
**Not Included in Meta-Analyses**				
Gallois et al. (2019) /France	N=168 mCRC (no data on colon or rectum cancers)<br><br>F (44%)<br>Race (no data reported)	During the chemotherapy (from start to 2 months)/CTCAE/<br><br>Moderate-to-severe overall chemotoxicity (26%):	In multivariate analysis, malnutrition was significantly associated with chemotherapy-related grade => clinical toxicities	Age itself was not a significant factor in chemotoxicity. Either functional status or nutritional status were

	Stages: Metastatic (100%).<br><br>Mean age: 75 (±5: 70-92)<br><br>5-FU single or combined with other agents (96%)/ Prospective study	nausea/vomiting (15%), diarrhea (8%), mucositis (4%), hand-foot skin reactions (0.6%, and alopecia (4%).	(malnutrition AOR 3.7; 1.7-8.4).<br>Included biomarkers such as lymphocytes, Hb, and albumin, but no variable of geriatric assessment.	significant factors of chemotoxicity.
Decoster et al. (2018)/ Belgium	N=252 mCRC<br>(colon only 75%)<br><br>F (38%)<br>Race (no data reported)<br>Stages: Metastatic (100%).<br><br>Mean age: 77 (±5: 69-91).<br><br>5-FU single or combined with other agents or capecitabine single or combined with other agents. / Prospective study	5-6 months after chemotherapy/physician evaluation/<br><br>Moderate-to-severe toxicity:<br>GI toxicity (13.6%), Cardiac toxicity (3.2%), and Vascular toxicity (35%).<br><br>(No available data of mild toxicity prevalence)	ECOG-PS (Eastern Cooperative Oncology Group-Performance status), geriatric assessment panels, fall history, comorbidities Nutritional status, ADL status were included.<br>However, only ECOG-PS significantly predicted chemotoxicity (no OR data).<br>No biomarkers added.	A higher risk of severe chemotoxicity was also associated with progression-free survival (PFS).
Djedi and Bouzid (2012)/Algeria	N=66 CRC patients<br>(no data on colon or rectum cancers)<br><br>F (47%)<br>Race (no data reported)<br>Stages (no data reported)<br><br>Mean age: 71 (±6; range 65-81, all ≥ 65 y.o.)<br><br>5-FU based agents (100%)/ Prospective study	Unknown time point post-chemotherapy /PROCTCAE/<br><br>Moderate-to-severe toxicity (50%).<br>-Non-hematological toxicity: neuropathy (5%), diarrhea (5%).<br>-Hematological toxicity: neutropenia (1%), anemia (6%).<br><br>(No available data of mild toxicity prevalence)	According to univariate and multivariate analyses, age (≤ 70 y.o.), geriatric assessment, anemia, and number of drugs used (mono vs poly-chemotherapy) were identified to be risk factors significantly associated with chemo-toxicity.<br>Beta or OR are not available.<br>No biomarkers	There is no report of the sensitivity or specificity of the prediction model.
Cespedes Feliciano et al. (2017)/USA	N= 533 patients<br>Colon only cancer (100%)<br><br>F (55.4%); Race White 61%, Black 8.8%, Hispanic/Latinx (13.15), Asian/Pacific Islander (16.2%), mixed races (0.7%)<br>Stages: II (13.8%), III (86.2%).	Between the first cycle initiation to the end of the first cycle (6 days-90 days after the date of initiation of chemotherapy)/Used ICD9 code EMR review/<br><br><br>Moderate-to-severe toxicity:	Muscle mass using clinically-acquired computed tomography (CT) scan to experience toxicities had twice the risk of adverse outcomes on FOLFOX: odds ratios (OR) and 95% Confidence Intervals	Lower muscle mass is associated with greater toxicity and poor chemotherapy adherence on FOLFOX. Many chemotherapy drugs are dosed on BSA; treatment may be better

	Mean age 58.7(±11:46-60)<br><br>5-FU-based agents (100%)/<br>Prospective study	-Non-hematological toxicity (no data reported)<br>-Hematological toxicity (33%).<br><br>(No available data of mild toxicity prevalence)	(95%CI) were OR=2.34 (95%CI: 1.04, 5.24; p-trend=0.03)	individualized if muscle mass is considered.
Grimes, C. (2022) /<br>USA	N=89 CRC<br>(no data on colon or rectum cancers)<br><br>F (58.4%)<br>Race (White 52.8%, Black 34.8%, others 12.3%); Stage: III (100%).<br><br>Mean age: 62 (no range)<br>5-FU single or combined with other agents (100%)/ Retrospective study	Incidence was examined after chemotherapy/no grading was used/<br><br>Moderate-to-severe chemotoxicity:<br>-Non-hematological toxicity: diarrhea (6.7%), nausea (5.6%), loss of appetite with dehydration (4.5%), thrombosis (3.4%), neuropathy (3.3%).<br>-Hematological toxicity (5.5%): leukocytosis (1.1%), Hospital admission due to toxicity (15.7%).	The primary risk factor was sarcopenia. Sarcopenia (measured with the CT scan), age, sex, and race were not associated with chemotoxicity.	African-American patients were less likely to receive adjuvant chemotherapy or continuous cancer treatments compared to Whites. Other measures, such as functional, cognitive, and nutritional frailty, may be associated with chemotoxicity.
Looijaard et al. (2020) /<br>Netherlands	N= 53 patients<br>Colon only cancer (100%)<br><br>F (45.4%)<br>Race White 61%, Black 8.8%, Hispanic/Latinx (13.15), Asian/Pacific Islander (16.2%), mixed races (0.7%)<br>Stages: II (1.9%), III (98.1%).<br><br>Mean age 70.9(±4: 67.5-73.5)<br><br>5-FU-based agents (50%) or capecitabine based (50%)/<br>Prospective study	After completion of chemotherapy/<br>Severe dose-limiting toxicity occurred in 52.8% of patients receiving chemotherapy.<br><br>Dose-limiting toxicity was defined as chemotherapy toxicity that led to a dose reduction or early discontinuation.<br><br>(No available data of mild toxicity prevalence)	After Bonferroni correction, no CT-based body composition measures were significantly associated with dose-limiting toxicity.	Future directions are suggested for a meta-analysis to elucidate the value of CT-based body composition measures to predict adverse outcomes in patients with CRC.

Note: ADL: activities of daily living; AOC: adjusted odds ratio; B: unstandardized beta coefficient; BMI: body mass index; BSA: body surface area; BUN/Cr: blood urea nitrogen/creatinine ratio; CA 19-9: cancer antigen 19-9; CAR: C-reactive protein and albumin ratio; CEA: carcinoembryonic antigen; CI: confidence interval; CRC: colorectal cancer; CRP: c-reactive protein; CT: computed tomography; CTCAE: common terminology criteria for adverse events; ECOG: eastern cooperative oncology group; ECOG-PS: eastern cooperative oncology group – performance score; EMR: electronic medical record; ET3: early grade III toxicity ; 5-FU: 5-Flouracil; FOLFOX: folinic acid, fluorouracil, and oxaliplatin; GI: gastrointestinal; GSP: glasgow prognostic score; Hb: hemoglobin; HEENT: head, eyes, ears, nose and throat; HR: hazard ratio; IADL: instrumental activities of daily living; ICD 9: international classification of diseases, ninth revision; mCRC: metastatic colorectal cancer; NLR: neutrophil to lymphocyte ratio; OR: odds ratio; PFS: progression free survival; PLR: platelet lymphocyte ratio; PLT: platelet count test; PRO-CTCAE: patient reported outcome – common terminology criteria for adverse events; RBC: red blood cell; SE: standard error; WBC: white blood cell.

Supplementary Figures

Supplementary Figure S1. Funnel Plots for Asymmetry Tests: Subgroups of Prevalences of Chemotoxicities.

A. Funnel Plot for Prevalence of Moderate-to-Severe Chemotoxicity

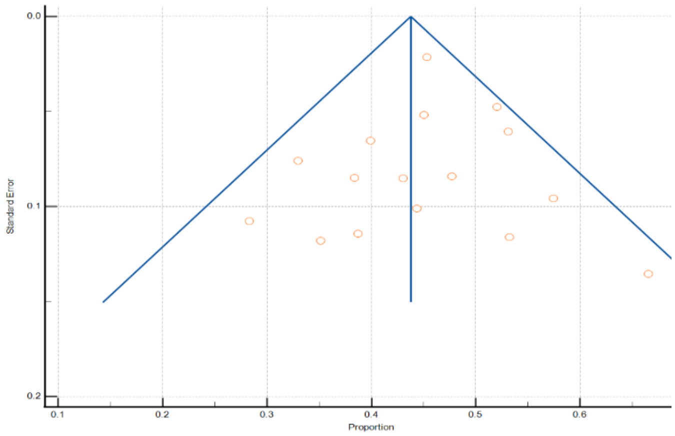

Non-Hematological Toxicity

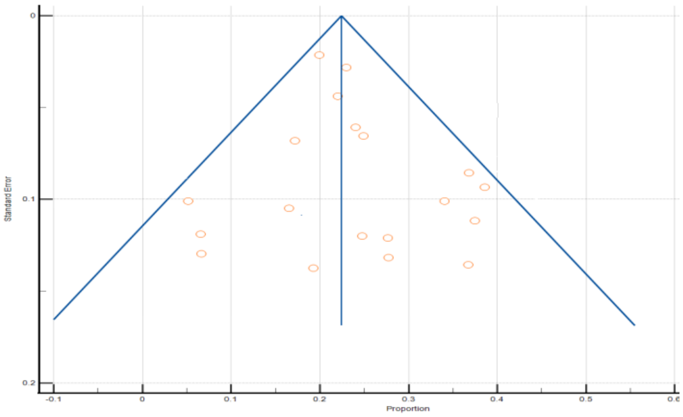

Hematological Toxicity

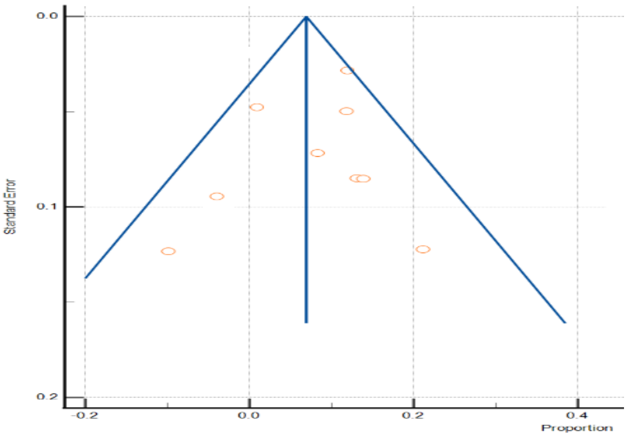

Neuropathy

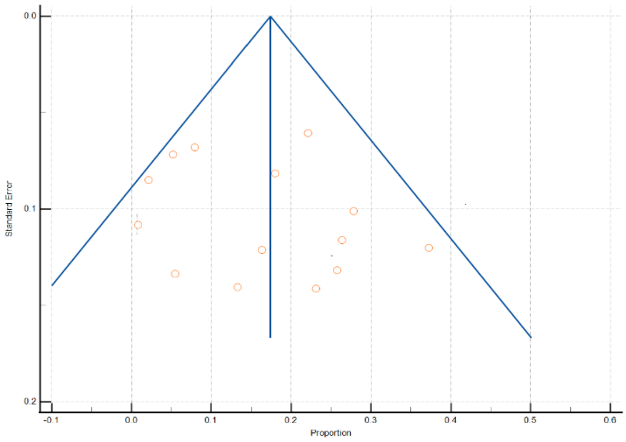

Neutropenia

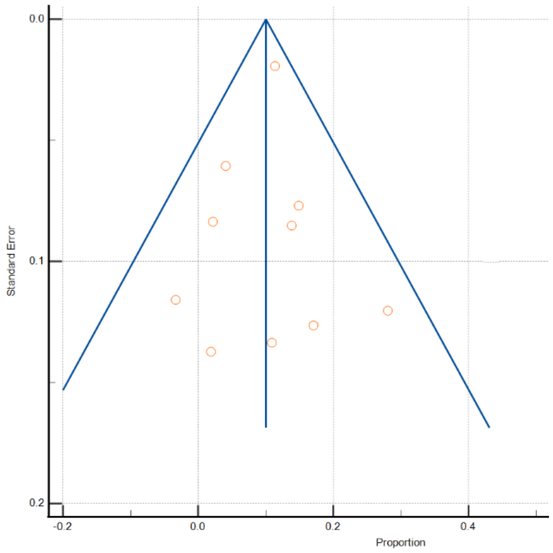

Nausea/Vomiting

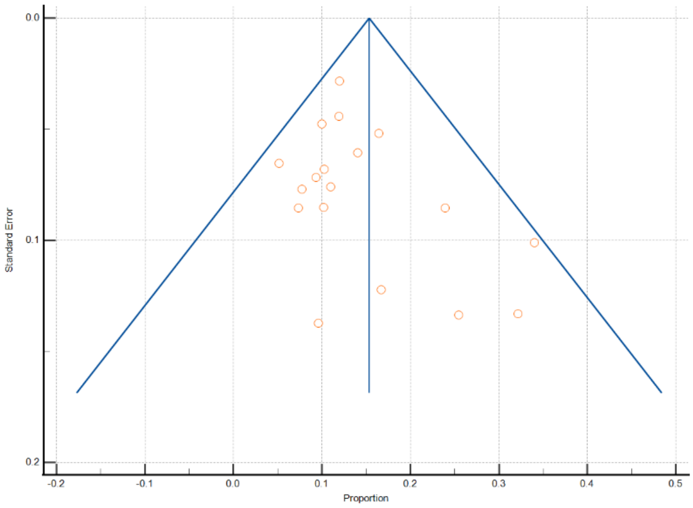

Diarrhea

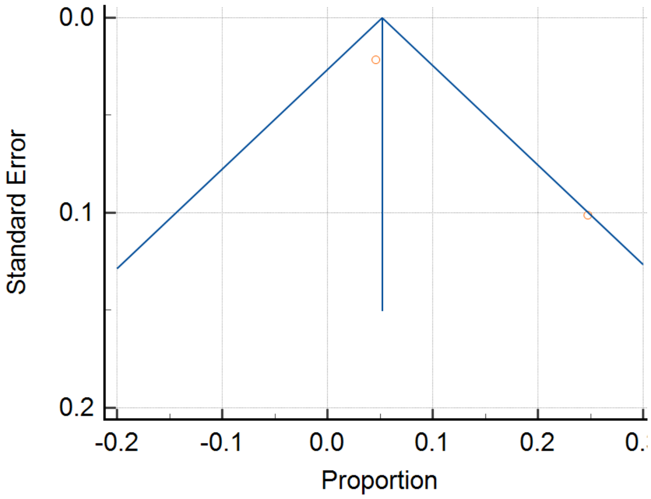

Leukocytosis

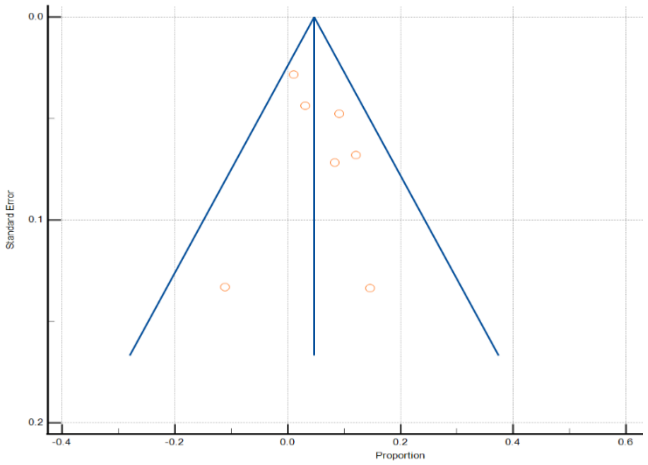

Fatigue

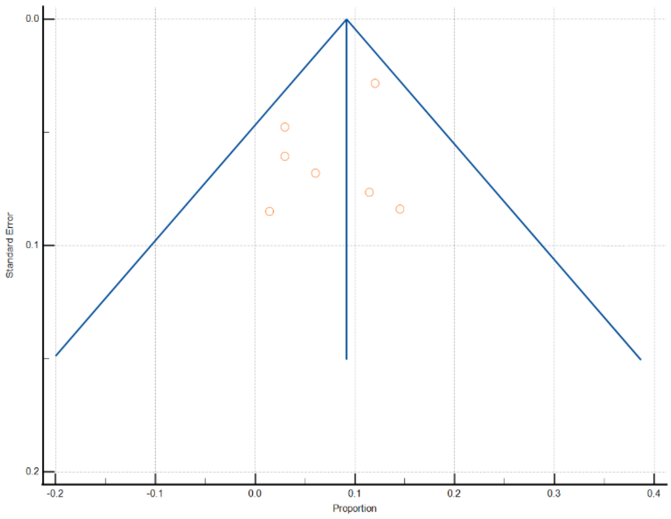

Anemia

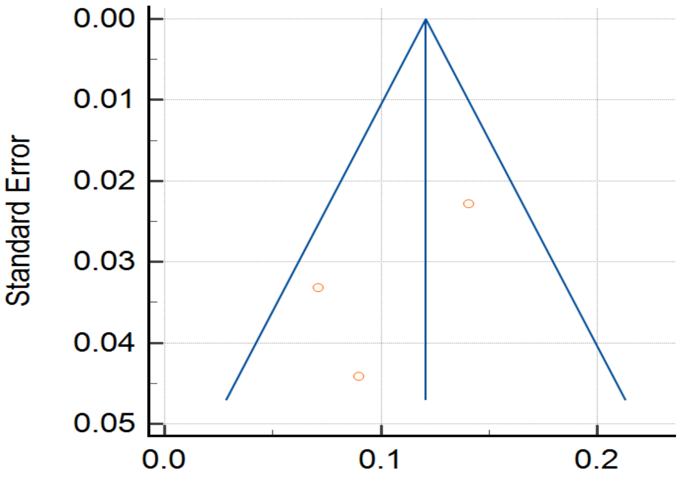

Leukopenia

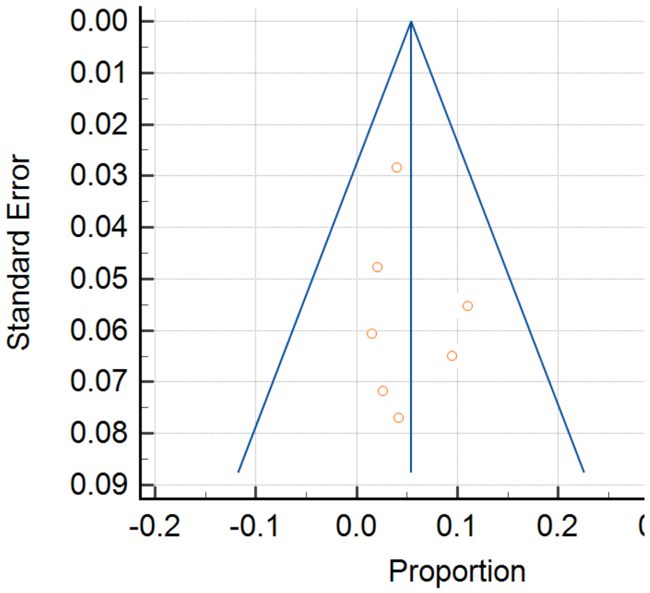

Mucositis/stomatitis

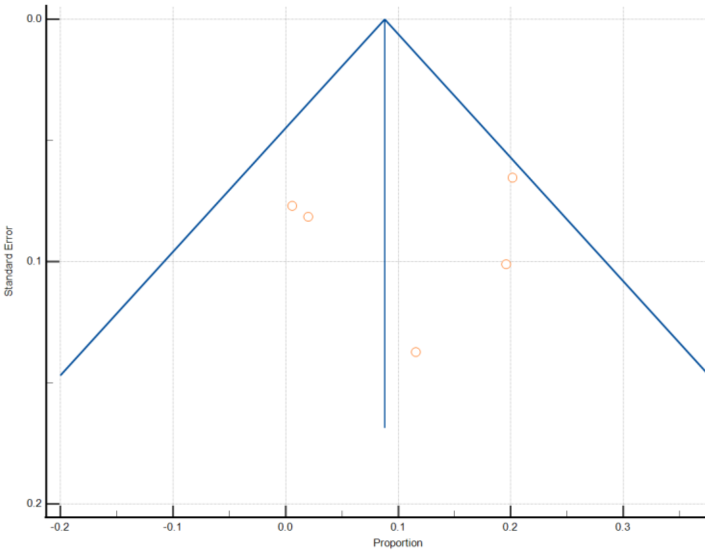

Hand-Foot-Syndrome

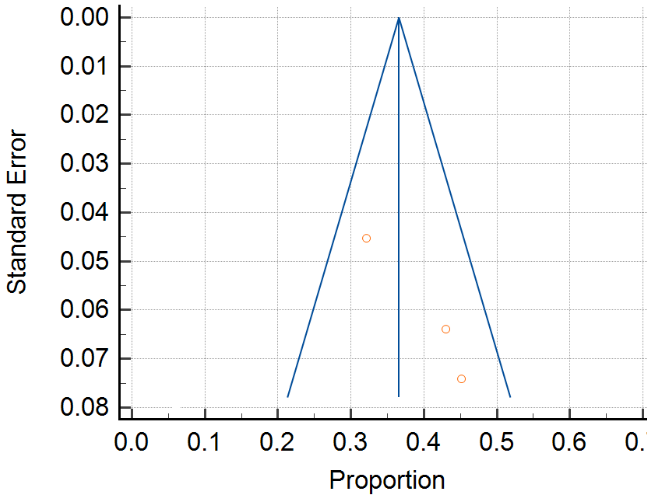

Abdominal Pain

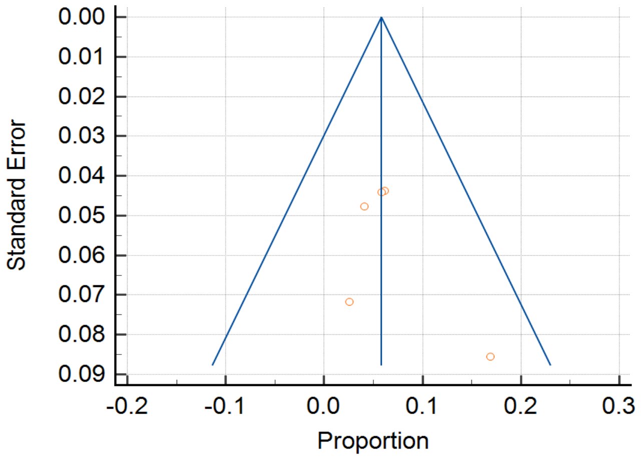

Anorexia

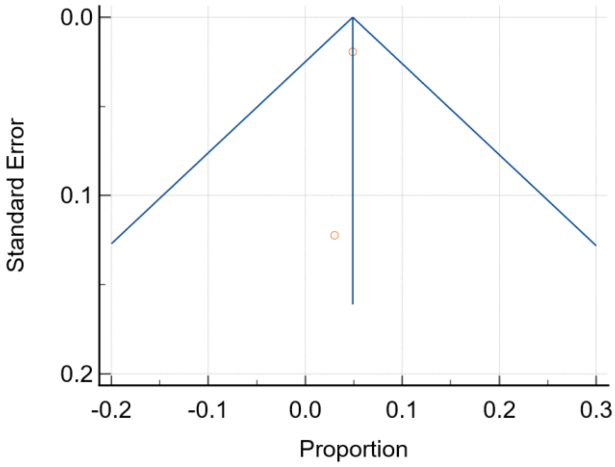

Coagulation Disorders

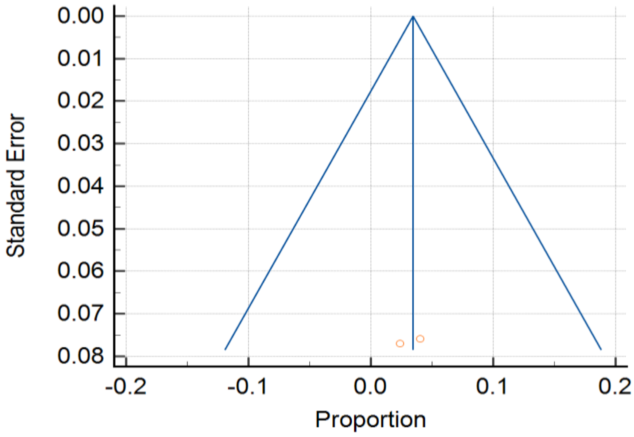

Constipation

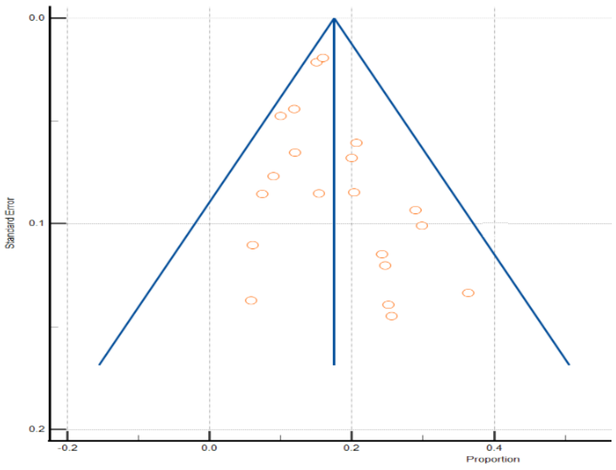

GI Toxicity

B. Funnel plot for the prevalence of mild chemotoxicity

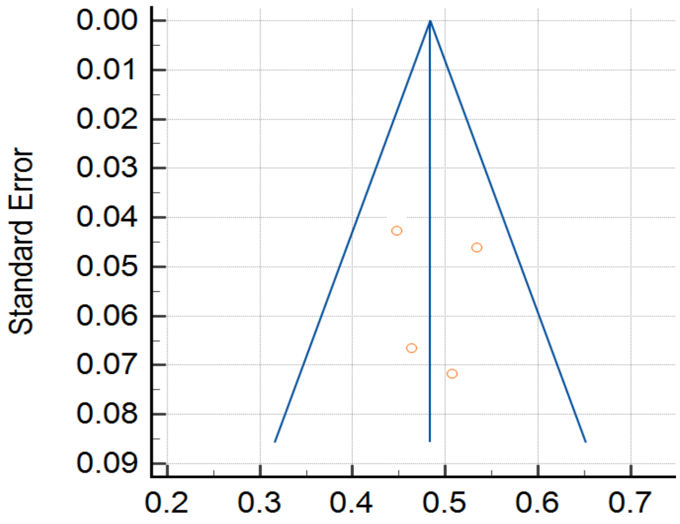

Diarrhea

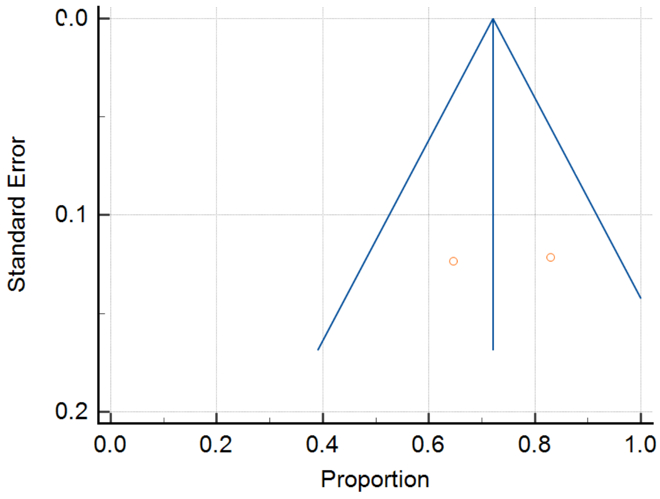

Hand-Foot-Syndrome

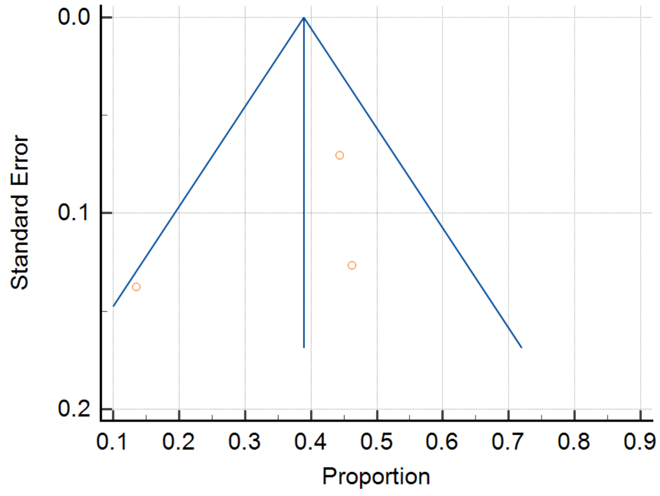

Anemia

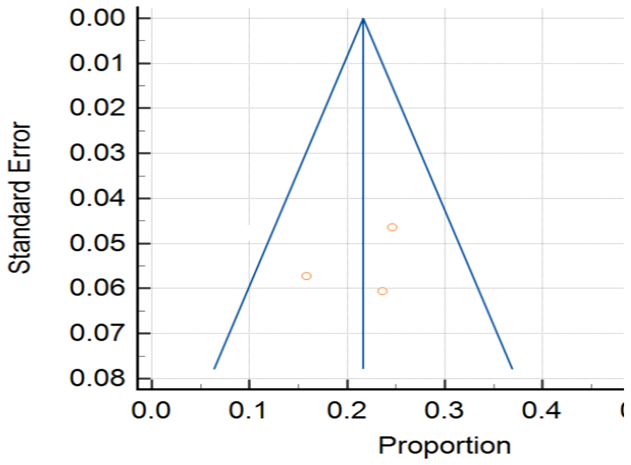

Mucositis/stomatitis

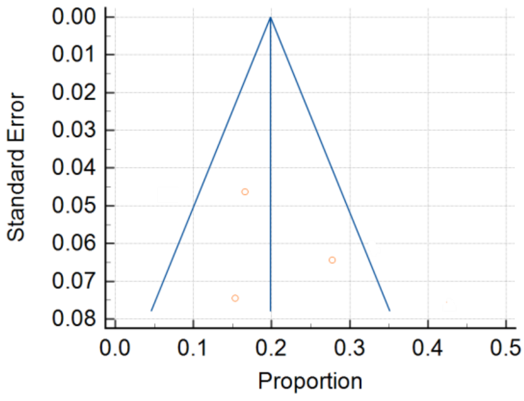

Neutropenia

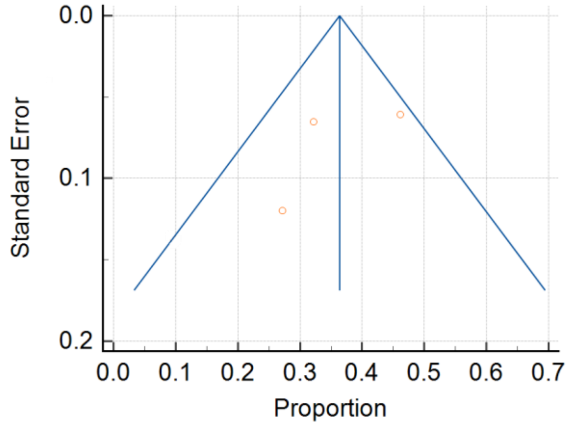

Nausea/Vomiting

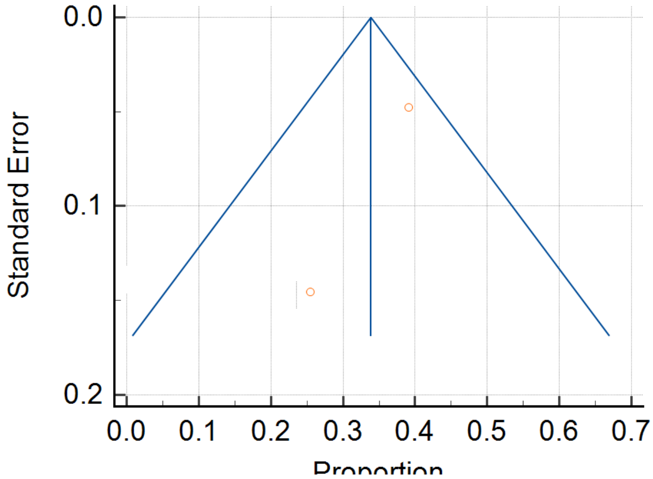

Fatigue

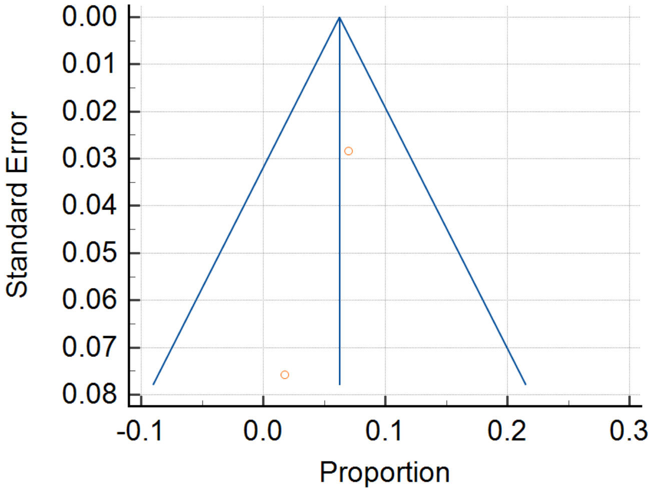

Coagulation disorders

Supplementary Figure S2.

Forest Plots for Pooled Prevalences of Chemotoxicities (Subgroups with Moderate-to-Severe Severity).

A. Moderate-to-Severe Abdominal Pain

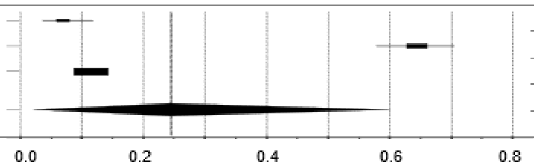

Study	Pooled Prevalence (%)	95% CI	Weight (%) Random
Garg et al. (2012)	6.93	3.63 to 11.80	10.49
Li et al. (2021)	64.37	57.86 to 70.52	14.11
Stein et al. (2016)	11.52	9.81 to 13.43	75.39
**Total: Random Effects**	**24.35**	2.07 to 60.15	100.00

B. Moderate-to-Severe GI Toxicity

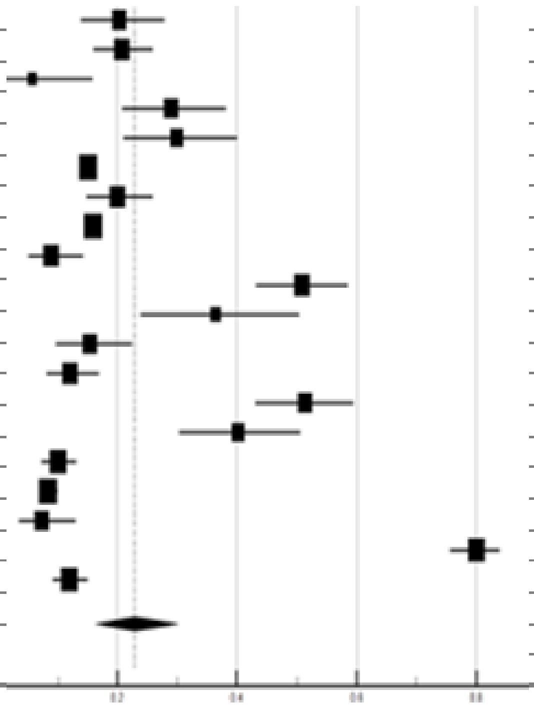

Study	Pooled Prevalence (%)	95% CI	Weight (%) Random
Ali et al. (2016)	20.29	13.92 to 27.97	4.96
Aparicio et al. (2016)	20.66	16.00 to 25.97	5.09
Backshall et al. (2011)	5.76	1.20 to 15.94	4.58
Barret et al. (2011)	28.94	20.83 to 38.18	4.91
Beukers et al. (2021)	29.89	21.02 to 40.04	4.86
Breton et al. (2021)	15.02	13.55 to 16.58	5.21
Feliu et al. (2022)	20.00	14.86 to 25.97	5.06
Folprecht et al. (2008)	15.90	14.54 to 17.34	5.22
Gallois et al. (2019)	8.92	5.08 to 14.29	5.01
Garg et al. (2012)	50.86	43.17 to 58.53	5.02
Hochster et al. (2007)	36.36	23.81 to 50.43	4.61
Karabulut et al. (2022)	15.32	9.74 to 22.47	4.96
Li et al. (2021)	12.01	8.13 to 16.89	5.07
Osterlund et al. (2007)	51.33	43.04 to 59.56	4.98
Retornaz et al. (2020)	40.20	30.37 to 50.65	4.86
Seymour et al. (2011)	10.00	7.36 to 13.19	5.14
Stein et al. (2016)	8.40	6.92 to 10.08	5.20
Tominga et al. (2016)	7.35	3.58 to 13.10	4.96
Watanabe et al. (2018)	80.05	75.62 to 84.00	5.13
Yamada et al. (2013)	11.91	9.23 to 15.04	5.16
**Total: Random Effects**	**22.87**	16.39 to 30.09	100.00

C. Moderate-to-Severe Neuropathy

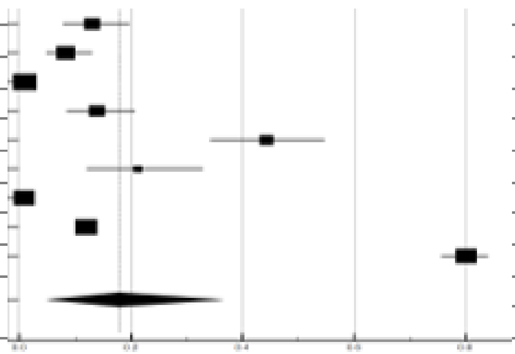

Study	Pooled Prevalence (%)	95% CI	Weight (%) Random
Ali et al. (2016)	13.04	7.91 to 19.82	11.07
Antonio et al. (2018)	8.29	4.81 to 13.11	11.12
Folprecht et al. (2008)	1.00	0.66 to 1.45	11.25
Karabulut et al. (2022)	13.86	8.56 to 20.80	11.07
Retornaz et al. (2020)	44.33	34.24 to 54.77	10.99
Sastre et al. (2012)	21.21	12.11 to 33.02	10.87
Seymour et al. (2011)	0.90	0.24 to 2.31	11.20
Stein et al. (2016)	12.01	10.25 to 13.94	11.24
Watanabe et al. (2018)	80.05	75.62 to 84.00	11.19
**Total: Random Effect**	**17.88**	4.91 to 36.47	100.00

D. Moderate-to-Severe Neutropenia

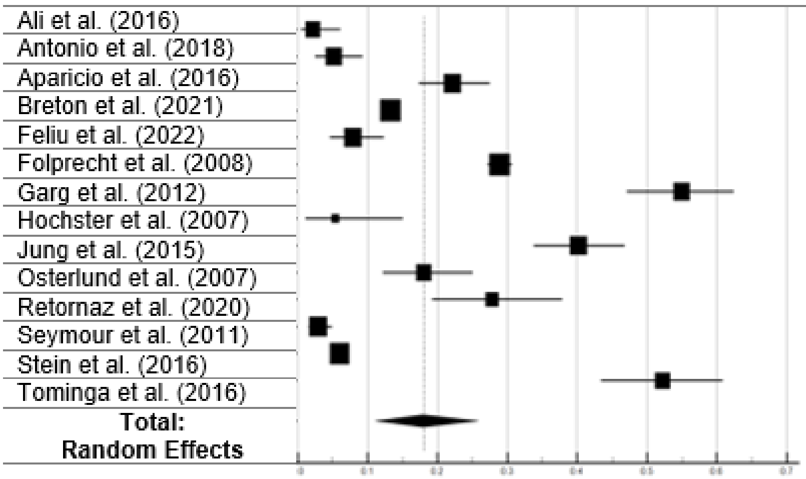

Study	Pooled Prevalence (%)	95% CI	Weight (%) Random
Ali et al. (2016)	2.17	0.45 to 6.22	7.05
Antonio et al. (2018)	5.18	2.51 to 9.32	7.15
Aparicio et al. (2016)	22.14	17.34 to 27.56	7.23
Breton et al. (2021)	13.24	11.84 to 14.73	7.41
Feliu et al. (2022)	7.90	4.67 to 12.35	7.18
Folprecht et al. (2008)	28.91	27.20 to 30.66	7.41
Garg et al. (2012)	54.91	47.18 to 62.47	7.12
Hochster et al. (2007)	5.45	1.13 to 15.12	6.54
Jung et al. (2015)	40.17	33.77 to 46.83	7.19
Osterlund et al. (2007)	18.00	12.21 to 25.09	7.08
Retornaz et al. (2020)	27.83	19.21 to 37.85	6.90
Seymour et al. (2011)	2.95	1.58 to 4.99	7.31
Stein et al. (2016)	6.00	4.75 to 7.46	7.39
Tominga et al. (2016)	52.20	43.47 to 60.83	7.04
**Total:**			
**Random Effects**	**17.91**	11.13 to 25.89	100.00

E. Moderate-to-Severe Nausea/Vomiting

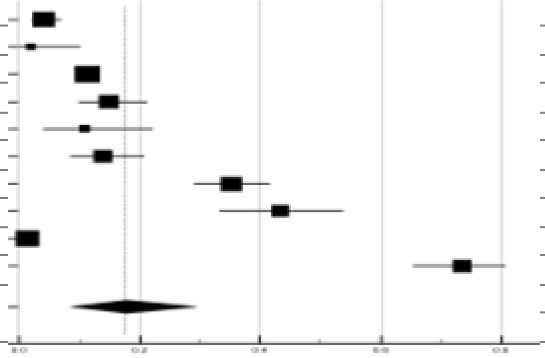

Study	Pooled Prevalence (%)	95% CI	Weight (%) Random
Aparicio et al. (2016)	4.05	2.04 to 7.14	10.19
Backshall et al. (2011)	1.92	0.048 to 10.25	9.47
Folprecht et al. (2008)	11.33	10.16 to 12.59	10.36
Gallois et al. (2019)	14.88	9.86 to 21.17	10.08
Hochster et al. (2007)	10.90	4.11 to 22.24	9.51
Karabulut et al. (2022)	13.86	8.56 to 20.80	10.01
Li et al. (2021)	35.19	29.07 to 41.69	10.16
Retornaz et al. (2020)	43.29	33.26 to 53.74	9.87
Stein et al. (2016)	1.44	0.85 to 2.26	10.34
Tominga et al. (2016)	73.52	65.28 to 80.72	10.01
**Total:**			
**Random Effects**	**17.78**	8.50 to 29.56	100.00

F. Moderate-to-Severe Diarrhea

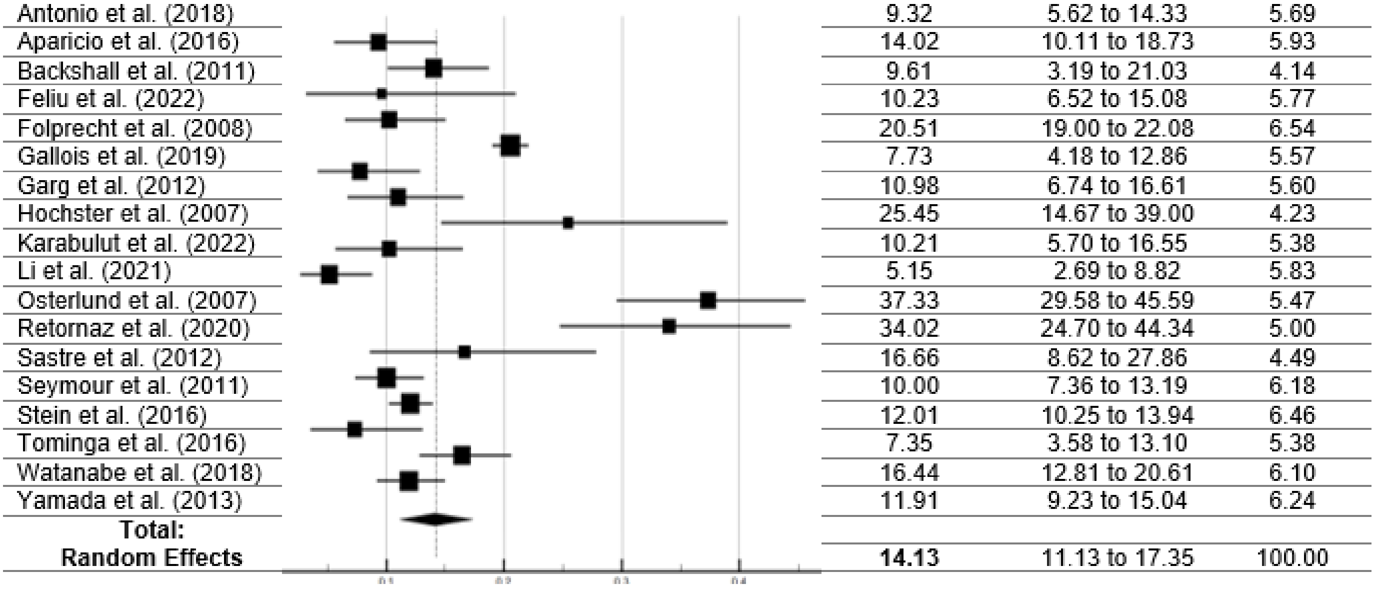

Study	Pooled Prevalence (%)	95% CI	Weight (%) Random
Antonio et al. (2018)	9.32	5.62 to 14.33	5.69
Aparicio et al. (2016)	14.02	10.11 to 18.73	5.93
Backshall et al. (2011)	9.61	3.19 to 21.03	4.14
Feliu et al. (2022)	10.23	6.52 to 15.08	5.77
Folprecht et al. (2008)	20.51	19.00 to 22.08	6.54
Gallois et al. (2019)	7.73	4.18 to 12.86	5.57
Garg et al. (2012)	10.98	6.74 to 16.61	5.60
Hochster et al. (2007)	25.45	14.67 to 39.00	4.23
Karabulut et al. (2022)	10.21	5.70 to 16.55	5.38
Li et al. (2021)	5.15	2.69 to 8.82	5.83
Osterlund et al. (2007)	37.33	29.58 to 45.59	5.47
Retornaz et al. (2020)	34.02	24.70 to 44.34	5.00
Sastre et al. (2012)	16.66	8.62 to 27.86	4.49
Seymour et al. (2011)	10.00	7.36 to 13.19	6.18
Stein et al. (2016)	12.01	10.25 to 13.94	6.46
Tominga et al. (2016)	7.35	3.58 to 13.10	5.38
Watanabe et al. (2018)	16.44	12.81 to 20.61	6.10
Yamada et al. (2013)	11.91	9.23 to 15.04	6.24
**Total:**			
**Random Effects**	**14.13**	11.13 to 17.35	100.00

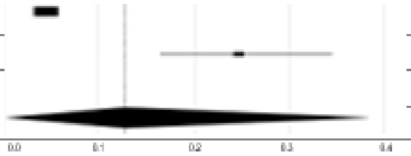

Study	G. Moderate-to-Severe Leukocytosis	Pooled Prevalence (%)	95% CI	Weight (%) Random
Breton et al. (2021)		4.61	3.77 to 5.57	51.30
Retornaz et al. (2020)		24.74	16.54 to 34.54	48.70
**Total:**				
**Random Effects**		**12.82**	0.43 to 38.24	100.00

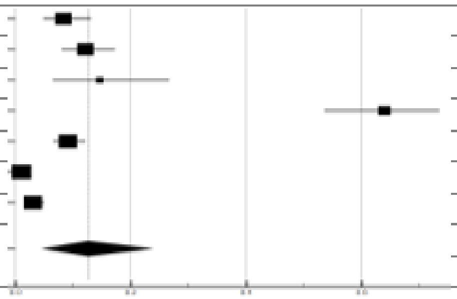

Study	H. Moderate-to-Severe Fatigue	Pooled Prevalence (%)	95% CI	Weight (%) Random
Antonio et al. (2018)		8.29	4.81 to 13.11	14.36
Feliu et al. (2022)		12.09	8.05 to 17.21	14.41
Hochster et al. (2007)		14.54	6.49 to 26.66	13.27
Retornaz et al. (2020)		63.91	53.53 to 73.42	13.90
Seymour et al. (2011)		9.09	6.57 to 12.17	14.63
Stein et al. (2016)		1.04	0.55 to 1.77	14.77
Tsuchihashi et al. (2018)		3.05	1.75 to 4.92	14.66
**Total:**				
**Random Effects**		**12.56**	4.60 o 23.69	100.00

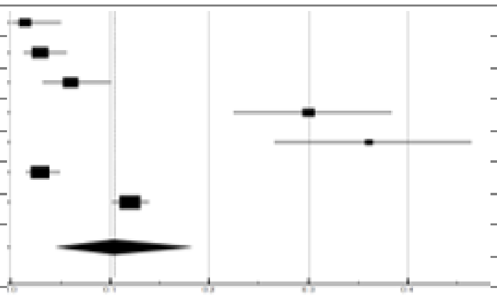

Study	I. Moderate-to-Severe Anemia	Pooled Prevalence (%)	95% CI	Weight (%) Random
Ali et al. (2016)		1.44	0.17 to 5.13	13.97
Aparicio et al. (2016)		2.95	1.28 to 5.73	14.50
Feliu et al. (2022)		6.04	3.25 to 10.11	14.35
Karabulut et al. (2022)		30.00	22.47 to 38.41	13.96
Retornaz et al. (2020)		36.08	26.57 to 46.46	13.54
Seymour et al. (2011)		2.95	1.58 to 4.99	14.72
Stein et al. (2016)		12.01	10.25 to 13.94	14.96
**Total:**				
**Random Effects**		**10.39**	4.54 to 18.26	100.00

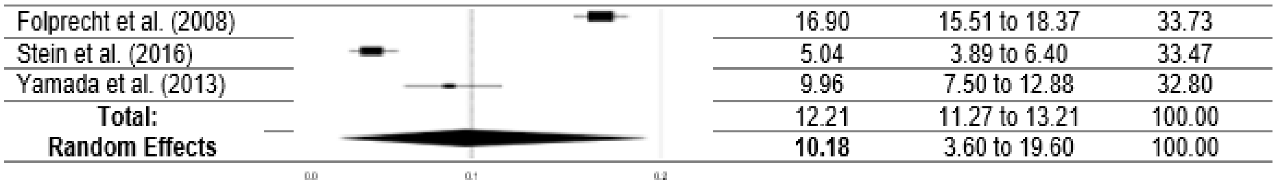

Study	J. Moderate-to-Severe Leukopenia	Pooled Prevalence (%)	95% CI	Weight (%) Random
Folprecht et al. (2008)		16.90	15.51 to 18.37	33.73
Stein et al. (2016)		5.04	3.89 to 6.40	33.47
Yamada et al. (2013)		9.96	7.50 to 12.88	32.80
**Total:**		12.21	11.27 to 13.21	100.00
**Random Effects**		**10.18**	3.60 to 19.60	100.00

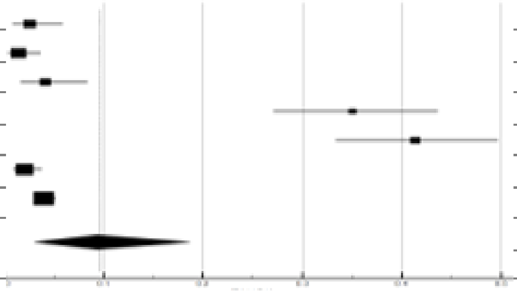

Study	K. Moderate-to-Severe Mucositis/Stomatitis	Pooled Prevalence (%)	95% CI	Weight (%) Random
Antonio et al. (2018)		2.59	0.84 to 5.94	14.22
Aparicio et al. (2016)		1.47	0.40 to 3.73	14.37
Gallois et al. (2019)		4.16	1.69 to 8.39	14.14
Karabulut et al. (2022)		35.03	27.08 to 43.64	14.00
Osterlund et al. (2007)		41.33	33.36 to 49.65	14.07
Seymour et al. (2011)		2.04	0.93 to 3.84	14.52
Stein et al. (2016)		4.00	2.98 to 5.24	14.68
**Total:**				
**Random Effects**		**9.35**	2.99 to 18.72	100.00

L. Moderate-to-Severe Hand-Foot Syndrome

Study	Pooled Prevalence (%)	95% CI	Weight (%) Random
Backshall et al. (2011)	11.53	4.35 to 23.44	18.76
Beukers et al. (2021)	19.58	12.22 to 28.88	19.84
Gallois et al. (2019)	0.59	0.011 to 3.27	20.42
Li et al. (2021)	20.17	15.21 to 25.90	20.65
Osterlund et al. (2007)	2.00	0.44 to 5.73	20.32
**Total: Random Effects**	**9.03**	1.83 to 20.89	100.00

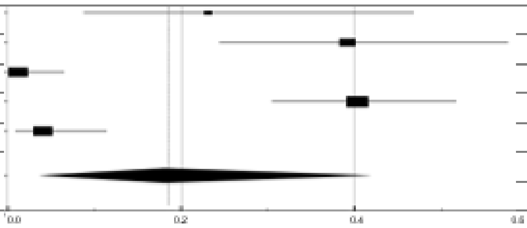

M. Moderate-to-Severe Anorexia

Study	Pooled Prevalence (%)	95% CI	Weight (%) Random
Antonio et al. (2018)	2.59	0.84 to 5.94	18.39
Seymour et al. (2011)	4.09	2.44 to 6.38	21.37
Tominga et al. (2016)	16.91	11.03 to 24.28	16.67
Tsuchihashi et al. (2018)	6.11	4.22 to 8.52	21.81
Yamada et al. (2013)	5.85	3.98 to 8.25	21.76
**Total: Random Effects**	**6.35**	3.72 to 9.61	100.00

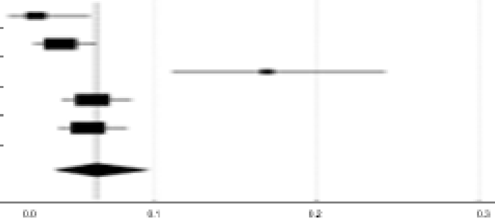

N. Moderate-to-Severe Coagulation Disorders

Study	Pooled Prevalence (%)	95% CI	Weight (%) Random
Folprecht et al. (2008)	4.91	4.12 to 5.79	97.57
Sastre et al. (2012)	3.03	0.36 to 10.52	2.43
**Total: Random Effects**	**4.89**	4.11 to 5.72	100.00

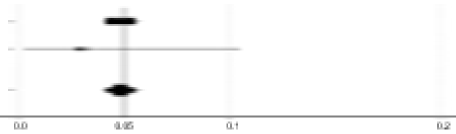

O. Moderate-to-Severe Constipation

Study	Pooled Prevalence (%)	95% CI	Weight (%) Random
Gallois et al. (2019)	2.38	0.65 to 5.98	49.27
Garg et al. (2012)	4.04	1.64 to 8.15	50.73
**Total: Random Effects**	**3.44**	1.77 to 5.63	100

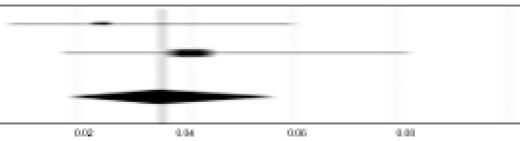

Supplementary Figure S3.

Forest Plots for Pooled Prevalence of Chemotoxicities (Subgroups with Mild Severity)

Study	Pooled Prevalence (%)	95% CI	Weight (%) Random
Antonio et al. (2018)	50.77	43.50 to 58.02	24.99
Garg et al. (2012)	83.23	76.82 to 88.47	24.95
Osterlund et al. (2007)	75.33	67.63 to 81.99	24.89
Seymour et al. (2011)	24.09	20.16 to 28.36	25.17
**Total: Random Effects**	**58.88**	28.80 to 85.69	100.00

A. Mild Diarrhea

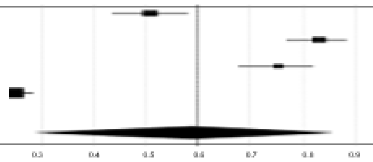

Study	Pooled Prevalence (%)	95% CI	Weight (%) Random
Backshall et al. (2011)	11.53	4.35 to 23.44	49.81
Osterlund et al. (2007)	90.00	84.04 to 94.29	50.19
**Total: Random Effects**	**51.74**	0.44 to 98.97	100.00

B. Mild Hand-Foot-Syndrome

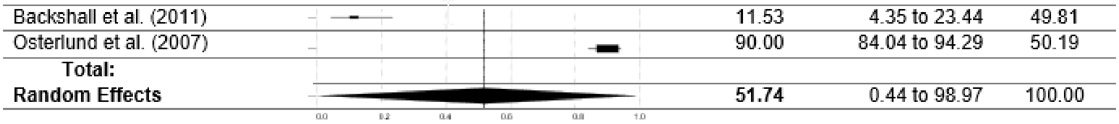

Study	Pooled Prevalence (%)	95% CI	Weight (%) Random
Aparicio et al. (2016)	81.91	76.81 to 86.31	33.51
Backshall et al. (2011)	13.46	5.58 to 25.78	32.92
Seymour et al. (2011)	17.04	13.65 to 20.89	33.57
**Total: Random Effects**	**36.50**	1.54 to 84.98	100.00

C. Mild Anemia

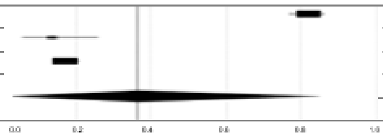

Study	Pooled Prevalence (%)	95% CI	Weight (%) Random
Aparicio et al. (2016)	23.61	18.68 to 29.13	33.36
Garg et al. (2012)	57.80	50.07 to 65.26	33.10
Seymour et al. (2011)	10.00	7.36 to 13.19	33.54
**Total: Random Effects**	**28.56**	7.21 to 57.01	100.00

D. Mild Mucositis/Stomatitis

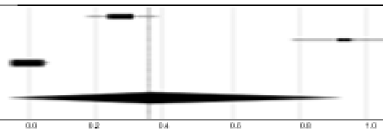

Study	Pooled Prevalence (%)	95% CI	Weight (%) Random
Aparicio et al. (2016)	32.84	27.28 to 38.78	33.31
Garg et al. (2012)	42.77	35.29 to 50.50	33.16
Stein et al. (2016)	2.48	1.69 to 3.50	33.53
**Total: Random Effects**	**22.44**	1.27 to 58.80	100.00

E. Mild Neutropenia

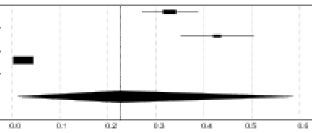

Study	Pooled Prevalence (%)	95% CI	Weight (%) Random
Aparicio et al. (2016)	46.12	40.07 to 52.26	33.65
Backshall et al. (2011)	9.61	3.19 to 21.03	32.46
Stein et al. (2016)	6.00	4.75 to 7.46	33.89
**Total: Random Effects**	**18.28**	0.56 to 52.28	100.00

F. Mild Nausea/Vomiting

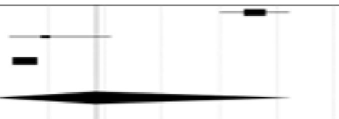

Study	Pooled Prevalence (%)	95% CI	Weight (%) Random
Backshall et al. (2011)	1.92	0.05 to 10.25	49.20
Seymour et al. (2011)	39.09	34.50 to 43.82	50.80
**Total: Random Effects**	**17.01**	0.54 to 63.68	100.00

G. Mild Fatigue

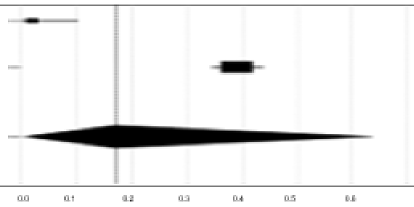

Study	Pooled Prevalence (%	95% CI	Weight (%) Random
Garg et al. (2012)	1.73	0.35 to 4.98	46.09
Stein et al. (2016)	6.96	5.61 to 8.52	53.91
**Total: Random Effects**	**4.33**	0.75 to 10.66	100.00

H. Mild Coagulation Disorders

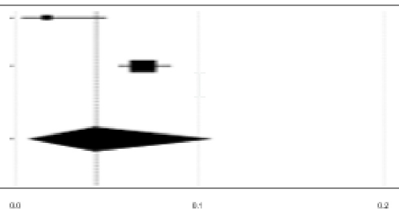

Supplement: Supplementary file 1 [file cancers-16-02597-s001.zip › cancers-3029769-supplementary.pdf]
